# Ventilator-Associated Pneumonia: The Role of Kidney–Lung Crosstalk and Renal Replacement Therapy, a Narrative Review

**DOI:** 10.3390/jcm15197570

**Published:** 2026-09-29

**Authors:** Luis Zamora, Bertrand Souweine, Jean-François Timsit, Claire Dupuis

**Affiliations:** 1 Department of Intensive Care Medicine, Clermont-Ferrand University Hospital (CHU Gabriel-Montpied), 63000 Clermont-Ferrand, France; lzamora@chu-clermontferrand.fr (L.Z.); bsouweine@chu-clermontferrand.fr (B.S.); 2 Medical and Infectious Diseases Intensive Care Unit, Bichat–Claude Bernard Hospital, Assistance Publique–Hôpitaux de Paris (AP-HP), 75018 Paris, France; jean-francois.timsit@aphp.fr; 3 INSERM UMR 1137, IAME, Université Paris Cité, 75018 Paris, France

**Keywords:** acute kidney injury, acute respiratory distress syndrome, kidney–lung crosstalk, ventilator-associated pneumonia, renal replacement therapy, critical illness, multiple organ dysfunction syndrome, mechanical ventilation

## Abstract

**Background/Objectives**: Multiple organ dysfunction syndrome (MODS) results from dynamic inter-organ interactions rather than isolated organ failures. Among these, kidney–lung crosstalk is a key determinant of outcomes in critically ill patients. Acute kidney injury (AKI) promotes pulmonary dysfunction through hemodynamic, inflammatory, endothelial, and metabolic mechanisms, while acute respiratory failure and mechanical ventilation may aggravate renal injury. Ventilator-associated pneumonia (VAP) may represent a clinical consequence of these interactions, although causality remains uncertain. Renal replacement therapy (RRT) may further influence kidney–lung crosstalk through several physiological mechanisms. To summarize current evidence on kidney–lung crosstalk, evaluate its potential contribution to VAP susceptibility, and examine the role of RRT in modulating these interactions. **Methods**: Narrative review of experimental, translational, and clinical studies investigating bidirectional kidney–lung interactions in critical illness. **Results**: Kidney-to-lung crosstalk involves fluid overload, venous congestion, immune activation, endothelial dysfunction, and metabolic disturbances. Lung-to-kidney signaling is driven by mechanical ventilation, ventilator-induced lung injury, hypoxemia, and hypercapnia. Clinical evidence is strongest for fluid overload, venous congestion, and mechanical ventilation, whereas endothelial dysfunction and uremic toxins remain supported mainly by experimental data. AKI-associated immune dysfunction and prolonged mechanical ventilation provide biologically plausible mechanisms linking AKI to VAP, but definitive causal evidence is lacking because of methodological limitations. RRT may influence these interactions through optimized fluid balance, correction of acid–base disorders, improved antimicrobial pharmacokinetics, and potentially extracorporeal immunomodulation. However, no randomized trial has demonstrated a direct reduction in VAP incidence attributable to RRT. **Conclusions:** Kidney–lung crosstalk is a major driver of organ dysfunction in critical illness, yet translation into targeted therapies remains limited. Current evidence supports integrated organ-protective strategies—including lung-protective ventilation, individualized fluid management, VAP prevention, and optimized antimicrobial therapy—while future advances will rely on biological phenotyping, biomarker-guided risk stratification, and robust causal inference approaches.

## 1. Introduction

Critical illness triggers a complex and dynamic systemic response involving inflammatory, immune, endocrine, and metabolic pathways that may ultimately culminate in multiple organ dysfunction syndrome (MODS) [1,2]. Historically viewed as the coexistence of independent organ failures, this progression is now understood to involve dynamic inter-organ interactions, organ crosstalk, through which an initial injury can induce or amplify dysfunction in distant organs via hemodynamic, inflammatory, neurohormonal, and metabolic pathways [3,4,5]. These interactions represent a fundamental framework for understanding MODS in intensive care medicine.

Among these networks, the kidney–lung axis occupies a particularly important position. In a landmark 2016 review, Husain-Syed et al. described the bidirectional mechanisms linking acute kidney injury (AKI) and acute pulmonary dysfunction through four major pathways: hemodynamic, immune-inflammatory, endothelial, and metabolic [3]. The principal mechanisms of this bidirectional crosstalk are summarized in Figure 1 [3,5,6]. Sullivan et al., 2025, subsequently expanded this framework by incorporating emerging evidence regarding extracellular vesicles and epigenetic inter-organ signaling pathways [7].

These kidney–lung interactions may contribute to the pathogenesis of ventilator-associated pneumonia (VAP), acting as predisposing factors, consequences, or amplifiers of organ dysfunction [8,9]. Within this context, renal replacement therapy (RRT) occupies a unique position. Beyond its established role as supportive therapy for severe AKI, RRT may influence kidney–lung crosstalk through effects on fluid balance, acid–base and metabolic homeostasis, inflammatory mediator clearance, and antimicrobial pharmacokinetics. However, whether these physiological effects translate into a reduced risk of VAP remains uncertain.

## 2. Objective

This narrative review provides an updated overview of kidney–lung crosstalk mechanisms, examines their potential contribution to VAP susceptibility, and critically evaluates the role of RRT in modulating these interactions. Particular attention is paid to distinguishing experimental evidence from clinical observations and to highlighting the strength of available evidence.

## 3. Methodology

A quasi-systematic literature search was conducted in PubMed without restriction on the publication period. The search strategy combined controlled vocabulary and free-text terms related to kidney injury, pulmonary injury, and interactions between the two organs. The following Boolean structure was used: (“acute kidney injury” OR “renal failure” OR “renal dysfunction” OR uremia) AND (“acute lung injury” OR “acute respiratory distress syndrome” OR “respiratory failure” OR “mechanical ventilation” OR “ventilator-associated pneumonia”) AND (“lung–kidney crosstalk” OR “kidney–lung crosstalk” OR “organ interaction” OR “distant organ injury” OR “renal replacement therapy” OR “fluid overload”). The flow-chart of the study selection is presented in Supplementary Figure S1. All publication types were considered eligible, including experimental studies, observational studies, randomized controlled trials, and reviews. Study selection was performed independently by two investigators (CD and LZ) through sequential screening of titles, abstracts, and full texts, with disagreements resolved by consensus. Additional references identified through Google Scholar and the reference lists of included articles were incorporated where relevant.

The conceptual figures were generated using ChatGPT (OpenAI, San Francisco, CA, USA; GPT-5.5) based on detailed prompts provided by the authors. The authors reviewed, edited, and validated all graphical elements and assume full responsibility for the scientific accuracy and content of the figures.

## 4. Pathophysiology from the Kidney to the Lung

### 4.1. Fluid Overload and Congestion

AKI is frequently accompanied by fluid overload resulting from impaired glomerular filtration and neurohormonal activation. Excess fluid accumulation promotes interstitial and alveolar edema, particularly in the presence of increased alveolar–capillary permeability [10,11]. Elevated central venous pressure (CVP) further worsens pulmonary congestion by impairing lymphatic drainage and increasing capillary hydrostatic pressure [12]. Collectively, these alterations reduce lung compliance, increase airway resistance, impair gas exchange, and contribute to prolonged dependence on mechanical ventilation [13,14].

Experimental studies support a direct role of AKI-associated fluid overload in pulmonary injury. In murine models of sepsis-induced AKI and renal ischemia–reperfusion, fluid accumulation following AKI increased alveolar permeability, lung wet-to-dry weight ratio, and pulmonary leukocyte infiltration independently of cardiac dysfunction [15,16]. These observations suggest that fluid overload is not merely a marker of disease severity but also contributes to cytokine-mediated endothelial activation and amplification of kidney–lung crosstalk [3,6,15].

The association between fluid overload and adverse respiratory outcomes is well documented clinically. In the SOAP study, including 3147 critically ill patients, positive cumulative fluid balance was independently associated with increased mortality among patients with AKI [17]. Legrand et al., 2013, showed that CVP independently predicted persistent AKI after adjustment for fluid balance and PEEP (OR 1.22 [1.08–1.39], *p* = 0.002), highlighting the central role of venous congestion [18]. In the FINNAKI cohort (n = 2901), fluid overload at RRT initiation was associated with higher 90-day mortality (OR 1.12 per excess liter, *p* < 0.001) [19]. Hayashi et al. and Silversides et al. confirmed these findings in independent cohorts [14,20]. Key clinical studies supporting this pathway are summarized in Table 1.

Venous congestion appears to be a major mechanistic link between renal and pulmonary dysfunction. Elevated CVP, whether related to fluid overload, high PEEP, or right ventricular (RV) dysfunction, reduces effective renal perfusion pressure and may impair glomerular filtration despite preserved systemic blood pressure [21,22]. RV failure, a frequent complication of severe ARDS, further amplifies this interaction by promoting renal congestion and AKI, while AKI-induced fluid overload and systemic inflammation increase RV afterload, creating a self-reinforcing cycle [23]. Vieillard-Baron et al., 2018, comprehensively described these cardiorenal–pulmonary interactions, whereas Beaubien-Souligny et al. introduced the VExUS score to quantify systemic venous congestion at the bedside, with promising implications for AKI prevention and management [23,24]. However, no randomized trial has yet demonstrated that targeted reduction in venous congestion improves renal outcomes.

Randomized controlled trials evaluating fluid management strategies provide complementary evidence (Supplementary Table S1). In patients with ARDS, the FACTT trial showed that a conservative fluid strategy increased ventilator-free days and reduced the need for RRT in secondary analyses [25]. By contrast, the CLASSIC and CLOVERS trials did not demonstrate a clear renal benefit of restrictive fluid strategies in unselected patients with septic shock [26,27]. More recently, the REVERSE-AKI pilot trial suggested a lower requirement for RRT among patients with established euvolemic AKI receiving protocolized deresuscitation, although these findings require confirmation [28]. Accordingly, the 2025 ESICM recommendations support active fluid deresuscitation during the recovery phase while acknowledging the limited high-quality evidence supporting this approach in the general ICU population [29].

Overall, fluid overload and venous congestion constitute key mechanisms linking AKI and pulmonary dysfunction. Although supported by strong biological plausibility and consistent observational evidence, establishing a definitive causal relationship remains challenging because of residual confounding and the complex interplay between illness severity, fluid administration, and organ dysfunction [11,30].

### 4.2. Immune–Inflammatory Pathway

AKI induces a profound systemic inflammatory response characterized by release of pro-inflammatory cytokines (IL-6, IL-1β, TNF-α), complement activation, and recruitment of innate immune cells [16,31], resulting from both increased mediator production and impaired renal clearance [32].

Experimental studies provide compelling evidence that inflammation represents an autonomous pathway of kidney–lung crosstalk. In a murine bilateral nephrectomy model, Hoke et al., 2007, demonstrated that AKI induces acute lung injury through a direct cytokine-mediated mechanism independent of fluid overload [15]. Subsequently, Faubel and Edelstein, 2016, identified circulating IL-6, IL-8, TNF-α, and CXCL1 as key molecular mediators linking renal injury to pulmonary dysfunction [6]. More recently, Mendes et al. expanded this framework to include extracellular vesicles and connexins as novel inter-organ mediators [32].

Within the lung, systemic inflammation promotes endothelial and epithelial activation, neutrophil recruitment, and increased vascular permeability [7,33]. Activated neutrophils release neutrophil extracellular traps (NETs), which contribute to tissue injury and may subsequently favor pulmonary immunoparalysis [33,34]. Complement activation through C3a and C5a further amplifies these processes [31]. Although isolating the specific contribution of AKI-induced inflammation in critically ill patients remains challenging, clinical observations support this biological framework. In patients with severe COVID-19, Ruault et al., 2025, reported an independent association between elevated IL-6 concentrations and early AKI, reinforcing the concept of a shared cytokine continuum linking renal and pulmonary injury [35]. However, the failure of anti-TNF therapies in sepsis illustrates the persistent gap between biological plausibility and clinical efficacy [32].

Evidence from immunomodulatory trials provides indirect support for the clinical relevance of this pathway (Supplementary Table S2) [36,37,38,39,40]. In COVID-19, dexamethasone in the RECOVERY trial reduced mortality and the need for RRT at day 28 [41]; tocilizumab demonstrated similar findings [42]. In non-COVID ARDS, methylprednisolone increased renal failure-free days in the ARDSNet LaSRS trial (22 vs. 18 days, *p* = 0.04) [43], and the CAPE COD trial demonstrated improved survival together with better organ function [44]. While these studies suggest that modulation of systemic inflammation may benefit both pulmonary and renal outcomes, a specific causal effect on kidney–lung crosstalk remains unproven.

Importantly, the initial hyperinflammatory phase of critical illness may evolve into acquired immunosuppression characterized by impaired neutrophil, monocyte, and macrophage function [45,46]. This transition from hyperactivation to immunoparalysis is increasingly recognized as a hallmark of persistent critical illness and may increase susceptibility to secondary infections, including VAP. Consequently, immune dysregulation may represent one of the most biologically plausible mechanisms linking AKI to pulmonary infection risk, although direct clinical evidence remains limited [3,5,6]. Key studies supporting the immune-inflammatory pathway are summarized in Table 2, while the principal cytokine networks are illustrated in Figure 2.

### 4.3. Endothelial Dysfunction, Glycocalyx Injury, and Microcirculatory Alterations

AKI is associated with systemic endothelial dysfunction, including upregulation of ICAM-1 and VCAM-1, glycocalyx degradation, and coagulation activation [49,50]. Glycocalyx disruption increases vascular permeability and amplifies inflammatory signaling [51], while concurrent coagulation and complement activation promote microvascular thrombosis and regional tissue hypoxia [49].

Experimental evidence suggests that this systemic endotheliopathy contributes to kidney–lung crosstalk. Faubel and Edelstein, 2016, proposed that endothelial activation mediates pulmonary injury through the dissemination of circulating inflammatory mediators released during AKI [6].

More recently, extracellular vesicles have emerged as additional mediators of inter-organ communication, capable of transporting inflammatory and procoagulant signals between injured organs [52].

Clinically, biomarkers of glycocalyx degradation, including syndecan-1 and heparan sulfate, correlate with disease severity and adverse outcomes in critically ill patients, although their specific association with pulmonary complications remains uncertain [51]. Therapeutic approaches aimed at preserving glycocalyx integrity have been proposed, but evidence remains limited and largely exploratory [53].

Overall, endothelial dysfunction represents a plausible mechanistic link between AKI and pulmonary injury. However, its relative contribution compared with hemodynamic congestion and systemic inflammation remains difficult to quantify, and direct evidence linking endothelial injury to clinically relevant respiratory outcomes is still lacking [3,6].

### 4.4. Acid–Base Disturbances and Uremic Toxins

Metabolic disturbances represent an additional component of kidney–lung crosstalk. Metabolic acidosis resulting from AKI increases respiratory drive and minute ventilation, impairs diaphragmatic function, and modulates systemic inflammatory responses [54]. Correction of severe acidemia may therefore theoretically improve both respiratory and renal physiology. However, clinical trials have yielded mixed results. In the BICAR-ICU and BICARICU-2 studies, intravenous sodium bicarbonate reduced the need for RRT in patients with severe AKI and metabolic acidosis, yet failed to demonstrate consistent benefits on mechanical ventilation duration or mortality [55,56]. These findings suggest that correction of acid–base abnormalities alone is insufficient to attenuate the pulmonary consequences of AKI, which are likely driven predominantly by hemodynamic, inflammatory, and endothelial mechanisms. Detailed results are summarized in Supplementary Table S3.

Acid–base homeostasis may also be influenced by fluid composition. Large-volume administration of normal saline can induce hyperchloremic metabolic acidosis, which has been associated with renal vasoconstriction, reduced glomerular filtration, and an increased risk of AKI [30]. Consistent with this concept, the SMART and SALT-ED trials demonstrated a reduction in major adverse kidney events with balanced crystalloids compared with normal saline [57,58], supporting the importance of chloride-restrictive strategies in critically ill patients.

Beyond acid–base disturbances, accumulation of uremic toxins may contribute to remote organ dysfunction. Experimental studies have shown that toxins such as indoxyl sulfate and p-cresyl sulfate impair innate immune responses through reduced neutrophil phagocytosis, altered antigen presentation, and disruption of epithelial barrier integrity [59,60]. Kaysi et al., 2026, further demonstrated dose-dependent increases in alveolar–capillary permeability following exposure to uremic toxins in vitro, suggesting a direct effect on pulmonary barrier function [61].

Although these observations provide strong biological plausibility, clinical evidence supporting a direct role of uremic toxins in pulmonary complications remains limited. Associations with respiratory outcomes, including VAP, have not been demonstrated in human studies, and routine measurement of circulating uremic toxins is not currently feasible in critical care practice.

Consequently, the contribution of metabolic disturbances and uremic toxins to kidney–lung crosstalk remains supported primarily by experimental data and appears less established than hemodynamic or inflammatory pathways [6].

Overall, fluid overload and venous congestion are supported by the strongest clinical evidence, whereas endothelial dysfunction and uremic toxins remain supported predominantly by mechanistic and experimental studies.

## 5. Pathophysiology from the Lung to the Kidney

### 5.1. Mechanical Ventilation and Renal Hemodynamics

Mechanical ventilation may adversely affect renal function through both hemodynamic and neurohormonal mechanisms. Increased intrathoracic pressure reduces venous return, cardiac output, and renal perfusion, while simultaneously increasing central venous pressure (CVP), thereby promoting renal venous congestion and reducing glomerular filtration pressure [21,22,62,63,64]. Importantly, these effects may occur even in the absence of systemic hypotension. Kumar et al. introduced the concept of a bidirectional pressure relationship between the ventilator and the kidney, whereby ventilatory settings directly influence renal hemodynamics [62]. Accordingly, recent recommendations from Meersch-Dini et al., 2026, recognize mechanical ventilation as an independent risk factor for AKI in critically ill patients [65]. However, the available evidence remains predominantly observational and does not establish a direct causal relationship.

### 5.2. Ventilator-Induced Lung Injury and Renal Tubular Damage

Beyond its hemodynamic consequences, mechanical ventilation may contribute to renal injury through ventilator-induced lung injury (VILI). Volutrauma, barotrauma, atelectrauma, and biotrauma trigger the systemic release of inflammatory mediators, including TNF-α, IL-1β, and IL-6, which promote endothelial activation, oxidative stress, tubular apoptosis, and disruption of renal cellular homeostasis [32,66,67,68,69]. This phenomenon extends the concept of organ crosstalk by linking pulmonary injury directly to remote organ dysfunction.

Experimental evidence supporting this pathway has been extensively reviewed by Curley et al., 2016 [66], while Mendes et al., 2024, highlighted the emerging role of extracellular vesicles as mediators of lung-to-kidney signaling [32].

Clinically, AKI is a frequent complication of ARDS. In a meta-analysis including more than 5000 patients, Antonucci et al., 2024, reported AKI rates ranging from 30% to 55%, with a twofold increase in hospital mortality among affected patients [70].

The strongest indirect evidence derives from the ARDSNet trial, in which lung-protective ventilation was associated with a lower incidence of renal dysfunction compared with conventional ventilation strategies [71].

Notably, elevated PEEP may contribute to renal injury through two complementary mechanisms: venous congestion and amplification of VILI through overdistension of relatively preserved lung regions. Together, these processes may create a self-perpetuating cycle of pulmonary inflammation and renal dysfunction [62,63].

### 5.3. Hypoxemia and Hypercapnia

Gas exchange abnormalities represent an additional pathway linking lung injury to renal dysfunction. Hypoxemia compromises oxygen delivery to the renal medulla, a region characterized by high metabolic demand and limited oxygen reserve, thereby promoting mitochondrial dysfunction, tubular injury, and apoptosis [72,73]. Although severe hypoxemia likely contributes to AKI in patients with respiratory failure, observational data suggest that the renal effects of mechanical ventilation cannot be explained solely by impaired oxygenation. In the PEEP-AKI-COVID study, elevated PEEP remained associated with AKI despite adjustment for oxygenation parameters, supporting a major role for intrathoracic pressure and venous congestion [64].

Hypercapnia may further aggravate renal dysfunction through direct and indirect mechanisms. Elevated arterial carbon dioxide tension increases renal vascular resistance via afferent arteriolar vasoconstriction and sympathetic activation, leading to reduced renal blood flow independently of systemic hemodynamics [32,73].

In parallel, activation of the renin–angiotensin–aldosterone system and vasopressin secretion promotes sodium and water retention. This adaptive response may subsequently worsen pulmonary congestion and respiratory mechanics, generating an additional feedback loop in which respiratory dysfunction promotes renal sodium retention, further amplifying lung injury [74]. Consistent with this concept, De Rosa et al. identified hypoxemia and hypercapnia as independent determinants of renal vulnerability in critically ill patients [73].

Overall, lung-to-kidney crosstalk involves intertwined hemodynamic, inflammatory, and gas-exchange pathways.

Mechanical ventilation and VILI are supported by the strongest experimental and clinical evidence, whereas the independent contributions of hypoxemia and hypercapnia remain less clearly established.

## 6. Kidney–Lung Crosstalk and VAP: A Critical Appraisal

### 6.1. AKI and Susceptibility to VAP

Several mechanisms support a biologically plausible association between AKI and increased susceptibility to ventilator-associated pneumonia (VAP). As discussed previously, AKI may promote pulmonary infection through acquired immunosuppression, impaired pulmonary host defenses, disruption of epithelial barrier integrity by uremic toxins, and prolonged exposure to mechanical ventilation resulting from fluid overload and delayed weaning.

The ADQI 28 consensus recognized AKI-induced immune dysfunction as a potential contributor to nosocomial infections, while emphasizing the limited clinical evidence supporting an independent effect of AKI on infection risk [48]. Experimental studies provide stronger support. Singbartl et al. demonstrated that AKI impairs pulmonary bacterial clearance and reduces alveolar macrophage phagocytic activity [75], whereas Doi and Rabb identified early alterations in pulmonary mucosal immunity following severe AKI [5]. Together, these findings suggest that AKI may directly compromise pulmonary immune competence, thereby facilitating secondary infections.

However, translating these mechanistic observations into clinical evidence remains challenging. Studies evaluating the association between AKI and VAP are hindered by substantial methodological heterogeneity, including variations in AKI definitions (RIFLE, AKIN, KDIGO) and VAP diagnostic criteria (CPIS, NHSN, ERS/ESICM) [76,77,78,79]. More importantly, duration of mechanical ventilation represents a major confounder because patients with AKI are more likely to require prolonged ventilatory support, independently increasing their risk of VAP [80].

Several epidemiological biases further complicate causal inference. Competing-risk bias may lead to underestimation of VAP incidence because the most severely ill patients may die before developing infection [80,81,82]. Immortal-time bias and confounding by indication related to heterogeneous preventive and therapeutic practices may also distort observed associations [82,83]. As highlighted by Wolkewitz et al. and Hazard et al., failure to account for these methodological issues can substantially alter estimates of VAP incidence and attributable risk [80,81].

Taken together, current evidence supports a biologically plausible relationship between AKI and VAP. Nevertheless, whether AKI independently increases VAP risk beyond its effects on illness severity and duration of mechanical ventilation remains unresolved.

### 6.2. VAP as a Driver of AKI

The relationship between AKI and VAP is likely bidirectional. Severe VAP may progress to pulmonary sepsis, triggering systemic inflammation, endothelial dysfunction, vasoplegia, microcirculatory alterations, and impaired renal perfusion [84,85,86]. Through these mechanisms, respiratory infection may contribute directly to the development of AKI as part of a broader syndrome of multi-organ dysfunction.

Treatment-related factors may further exacerbate renal injury. Nephrotoxic antimicrobials frequently used in severe VAP, including aminoglycosides and vancomycin, constitute an additional pathway linking pulmonary infection to AKI [87]. Consequently, both the infectious process itself and its management may contribute to renal dysfunction.

Clinical observations support this concept. In a meta-analysis of ARDS patients, Antonucci et al. reported AKI rates ranging from 30% to 55%, with AKI independently associated with increased hospital mortality [70]. Similarly, Peerapornratana et al. identified pulmonary sepsis as one of the leading causes of ICU-acquired AKI [84]. However, as in the AKI-to-VAP relationship, disentangling causality from shared disease severity remains difficult. Patients who develop severe VAP often exhibit greater baseline physiological derangement, making it challenging to determine whether AKI represents a direct consequence of pulmonary infection or simply a marker of critical illness severity [31].

Overall, available evidence suggests that AKI and VAP participate in a self-reinforcing cycle mediated by inflammation, immune dysregulation, prolonged mechanical ventilation, and organ dysfunction. While the biological rationale for this bidirectional interaction is compelling, robust clinical studies specifically designed to evaluate causal pathways remain lacking.

## 7. The Role of RRT in Kidney–Lung Crosstalk

RRT may influence kidney–lung crosstalk through several complementary mechanisms, including fluid balance control, correction of metabolic disturbances, modulation of inflammatory mediators, and optimization of antimicrobial exposure. However, despite strong physiological rationale, no randomized trial has demonstrated a direct reduction in VAP incidence attributable to RRT. Current evidence therefore supports an indirect modulatory role rather than a proven effect on pulmonary infectious outcomes. The principal mechanisms and corresponding levels of evidence are summarized in Figure 3.

### 7.1. Fluid Balance Control

Among the proposed mechanisms, fluid management is supported by the strongest clinical evidence [65,88]. By enabling precise and continuous fluid removal, CRRT may reduce pulmonary congestion, improve lung mechanics, and facilitate liberation from mechanical ventilation [14]. Because fluid overload and venous congestion represent key drivers of kidney–lung crosstalk, this pathway is biologically plausible and clinically relevant.

Nevertheless, interventional evidence remains limited. In the GO NEUTRAL trial, a hemodynamically guided neutral fluid balance strategy during CRRT was feasible and safe but did not improve mortality or duration of mechanical ventilation [89]. These findings suggest that while RRT effectively controls fluid balance, its impact on clinically meaningful respiratory outcomes remains uncertain.

### 7.2. Acid–Base Disorders

RRT rapidly corrects metabolic acidosis and associated electrolyte disturbances, potentially reducing respiratory drive, diaphragmatic workload, and the adverse physiological consequences of severe acidemia [54,90]. However, available clinical data do not support a major effect on pulmonary outcomes. Consistent with this observation, the BICARICU-2 trial demonstrated a reduction in RRT requirements following bicarbonate therapy without significant effects on mechanical ventilation duration or mortality [56]. Consequently, acid–base correction should be regarded as an important supportive intervention rather than a demonstrated strategy for reducing VAP risk or altering kidney–lung crosstalk.

### 7.3. Immunomodulation: Strong Plausibility, Limited Clinical Evidence

Extracorporeal cytokine removal has attracted considerable interest as a means of attenuating systemic inflammation. Proposed approaches include convective and diffusive clearance as well as adsorption using dedicated membranes and hemoadsorption devices [91,92,93,94]. However, clinical results have been disappointing. In the Clic-AKI study, Lumlertgul et al., 2021, demonstrated measurable removal of IL-6 and IL-8 using EMiC2 membranes without sustained reductions in circulating cytokine concentrations, suggesting that ongoing cytokine production exceeds extracorporeal clearance capacity [91,92]. This limitation reflects the “peak concentration hypothesis” originally proposed by Ronco et al. [91,92]. Similarly, systematic reviews have not demonstrated consistent benefits on mortality or infection-related outcomes [94]. Adsorptive technologies such as AN69-ST, oXiris^®^, and CytoSorb^®^ may transiently improve hemodynamic parameters, but evidence supporting reductions in mortality, AKI progression, or VAP incidence remains limited and heterogeneous [88,91]. Moreover, the non-selective removal of both harmful and protective mediators remains a theoretical concern. At present, extracorporeal immunomodulation should be considered investigational rather than standard therapy.

### 7.4. RRT and Antibiotic Pharmacokinetics

Unlike immunomodulation, the influence of RRT on antibiotic pharmacokinetics is well established and directly relevant to clinical practice. Clearance of hydrophilic antimicrobials, including β-lactams, vancomycin, and colistin, is substantially altered by RRT and depends on modality, effluent flow rate, membrane characteristics, protein binding, and molecular weight [95,96]. The DALI study demonstrated that more than 40% of critically ill patients failed to achieve pharmacodynamic targets for β-lactam therapy when dosing was not appropriately adjusted, leading to higher rates of treatment failure [97]. Current dosing algorithms incorporate RRT-specific parameters, and therapeutic drug monitoring is increasingly recommended, particularly for vancomycin using AUC/MIC-guided approaches [96,98].

Although this mechanism does not directly modify kidney–lung crosstalk, optimization of antibiotic exposure may indirectly influence pulmonary outcomes by improving VAP treatment and limiting both underexposure and toxicity.

### 7.5. Timing of RRT Initiation

The potential impact of RRT timing on pulmonary outcomes remains uncertain. Among major randomized trials evaluating early versus delayed RRT initiation (Supplementary Table S4), only the single-center ELAIN trial reported a reduction in mechanical ventilation duration with earlier RRT initiation [99]. This finding was not reproduced in AKIKI, IDEAL-ICU, or STARRT-AKI [100,101,102]. Differences in patient populations, disease severity, and initiation thresholds are likely to explain these discrepant results. Importantly, none of these studies was designed to evaluate VAP incidence or kidney–lung crosstalk as a primary endpoint. Accordingly, current evidence does not support early RRT initiation solely to improve respiratory outcomes, and initiation decisions should continue to rely on established clinical indications and guideline recommendations [65,103].

Among the mechanisms through which RRT may influence kidney–lung interactions, fluid balance optimization and antimicrobial pharmacokinetic management are supported by the strongest clinical evidence, whereas extracorporeal immunomodulation remains investigational (Table 3).

### 7.6. Experimental and Clinical Evidence Regarding the Role of Renal Replacement Therapy in Kidney–Lung Crosstalk

The available evidence indicates that the impact of RRT on kidney–lung crosstalk varies according to the underlying biological mechanism. Among the proposed pathways, fluid balance optimization and antimicrobial pharmacokinetic management are supported by the strongest clinical evidence and constitute the most relevant mechanisms in current practice. In contrast, extracorporeal immunomodulation, endothelial protection, and removal of uremic toxins remain supported predominantly by experimental or physiological studies, with little evidence of clinically meaningful benefit. Similarly, although correction of acid–base disturbances is an essential supportive function of RRT, its specific contribution to attenuating kidney–lung crosstalk has not been demonstrated.

Overall, current evidence supports an indirect modulatory role of RRT rather than a disease-modifying therapy targeting kidney–lung crosstalk. No randomized trial has shown that RRT reduces the incidence of ventilator-associated pneumonia or other pulmonary complications beyond its established supportive effects. Table 3 summarizes the principal experimental and clinical evidence for each proposed mechanism, highlights the corresponding level of evidence, and identifies the main knowledge gaps that should be addressed in future studies.

## 8. Clinical Implications

### 8.1. Lung-Protective Ventilation

Lung-protective ventilation remains a cornerstone strategy for limiting kidney–lung crosstalk. By reducing VILI and the systemic release of inflammatory mediators implicated in AKI, protective ventilation may attenuate both pulmonary and renal injury [66,71]. Current ATS/ESICM/SCCM guidelines recommend tidal volumes of approximately 6 mL/kg predicted body weight and plateau pressures ≤ 30 cmH_2_O in patients with ARDS [71]. By limiting biotrauma while avoiding excessive PEEP-related venous congestion, lung-protective ventilation should be regarded as both a lung- and kidney-protective intervention.

### 8.2. Individualized Fluid Management

Among currently available interventions, individualized fluid management likely represents the most effective strategy for mitigating kidney–lung interactions. Fluid overload promotes pulmonary edema, impaired gas exchange, venous congestion, right ventricular dysfunction, and progression of AKI, whereas excessive fluid removal may compromise organ perfusion [11,23,65,103]. Fluid management should therefore be tailored to the patient’s hemodynamic profile, respiratory status, and renal function [14,19,20,107]. When clinically indicated, RRT can facilitate controlled fluid removal, although current evidence does not support its use solely to improve respiratory outcomes [25,65,103,107,108]. Balanced crystalloids should generally be preferred over normal saline because of their more favorable renal and acid–base effects [57,58].

### 8.3. Prevention of VAP

Because no evidence supports a direct protective effect of RRT against VAP, prevention should rely on established evidence-based bundles. These include minimizing the duration of mechanical ventilation, optimizing sedation, promoting early mobilization, maintaining airway hygiene, and limiting unnecessary exposure to broad-spectrum antibiotics [108]. Beyond reducing pulmonary complications, these measures may indirectly protect renal function by preventing pulmonary sepsis and subsequent multi-organ dysfunction.

### 8.4. Optimization of Antimicrobial Therapy During RRT

RRT substantially alters the pharmacokinetics of many antimicrobial agents. Therapeutic drug monitoring and individualized dosing strategies accounting for RRT modality, membrane characteristics, and effluent flow rates should therefore be incorporated into routine practice, particularly for antibiotics with narrow therapeutic windows [106,108]. Optimizing antimicrobial exposure represents an essential component of integrated kidney–lung management and may contribute to improved outcomes in patients with severe pulmonary infections receiving RRT.

## 9. Research Gaps and Future Directions

Despite substantial advances in the understanding of kidney–lung crosstalk, important knowledge gaps remain. Most available evidence derives from observational studies, limiting causal inference. Future research should incorporate advanced causal methodologies, including target trial emulation, competing-risk analyses, multi-state models, and time-dependent approaches, particularly for outcomes such as VAP that are strongly influenced by extubation and mortality [80,81,109,110,111].

A second challenge relates to the biological heterogeneity of critical illness. Emerging evidence supports the existence of distinct ARDS and sepsis subphenotypes with differential inflammatory responses, organ dysfunction patterns, and treatment responsiveness [112,113]. Whether susceptibility to kidney–lung crosstalk varies across these biological phenotypes remains largely unknown.

Biomarkers of immune dysfunction, endothelial injury, glycocalyx degradation, and monocyte HLA-DR expression may help identify patients at increased risk of AKI-associated immunoparalysis and secondary pulmonary infections. Integrating such biomarkers into future studies may improve risk stratification and facilitate more personalized therapeutic approaches.

Finally, future interventional trials should move beyond short-term physiological endpoints and prioritize patient-centered outcomes, including duration of mechanical ventilation, functional recovery, quality of life, long-term kidney function, and post-intensive care syndrome [114].

## 10. Conclusions

Kidney–lung crosstalk is a central determinant of MODS in critically ill patients. Although its pathophysiological mechanisms are now well characterized experimentally, their clinical implications—particularly regarding VAP susceptibility—remain incompletely understood. The AKI–VAP relationship is biologically plausible and experimentally supported, but definitive causal demonstration is constrained by MV duration bias, competing risks, illness severity confounding, and other methodological challenges inherent to ICU research.

RRT may modulate kidney–lung interactions, principally through fluid balance control, metabolic correction, and antibiotic pharmacokinetic optimization, without demonstrated direct protection against VAP. Integrated kidney–lung protective strategies remain the most evidence-based approach: lung-protective ventilation, individualized fluid management with careful fluid selection, VAP prevention bundles, and pharmacokinetically optimized antimicrobial therapy.

Future advances in biological phenotyping, biomarker-guided risk stratification, extracorporeal therapy design, and causal inference methodologies may ultimately enable a more personalized approach to kidney–lung crosstalk and its infectious consequences in critically ill patients.

## Figures and Tables

**Figure 1:**
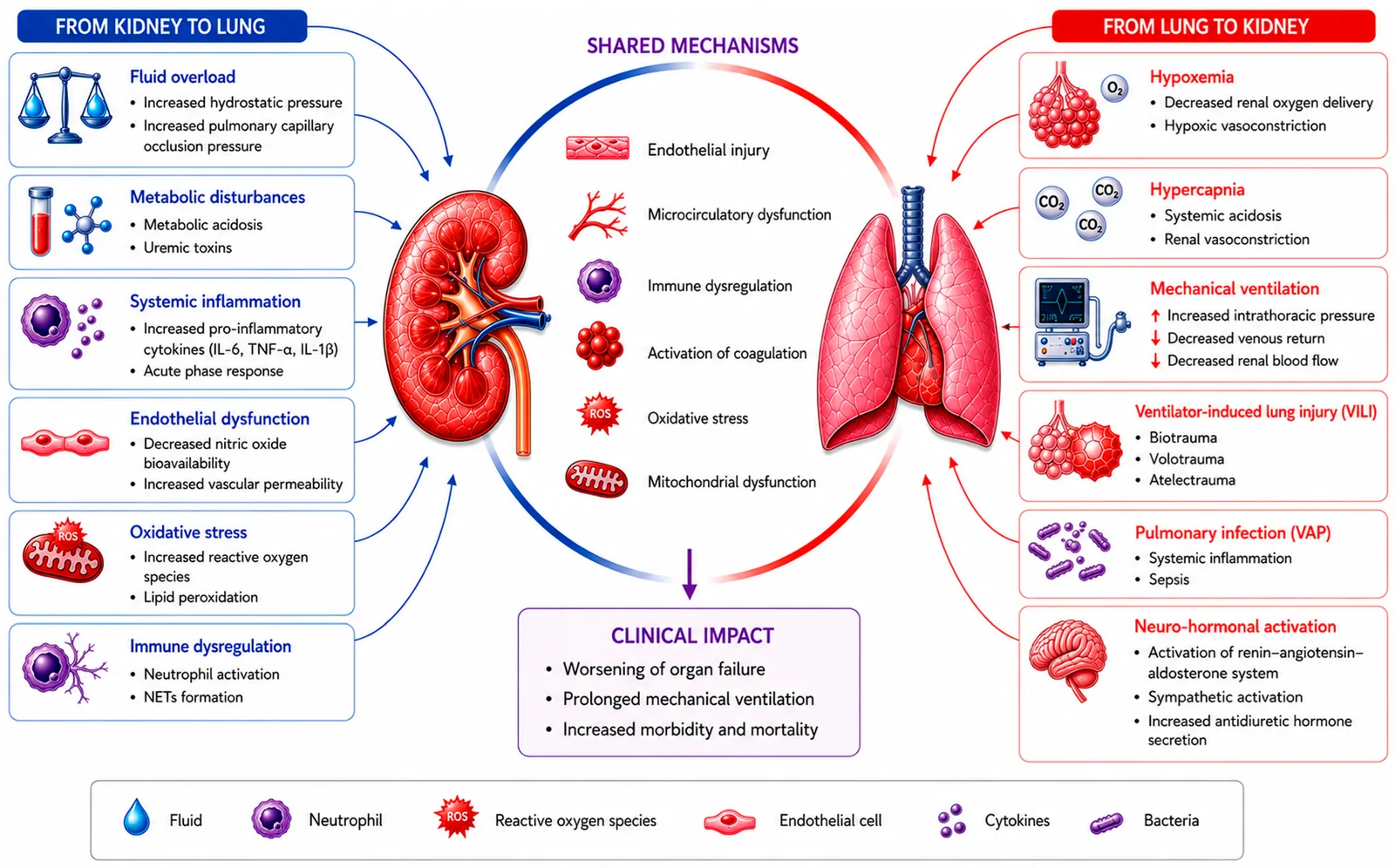
General overview of kidney–lung crosstalk and key interactions during critical illness. ADH: antidiuretic hormone; AKI: acute kidney injury; IL: interleukin; NETs: neutrophil extracellular traps; NO: nitric oxide; RAAS: renin–angiotensin–aldosterone system; ROS: reactive oxygen species; TNF-α: tumor necrosis factor alpha; VAP: ventilator-associated pneumonia; VILI: ventilator-induced lung injury.

**Figure 2:**
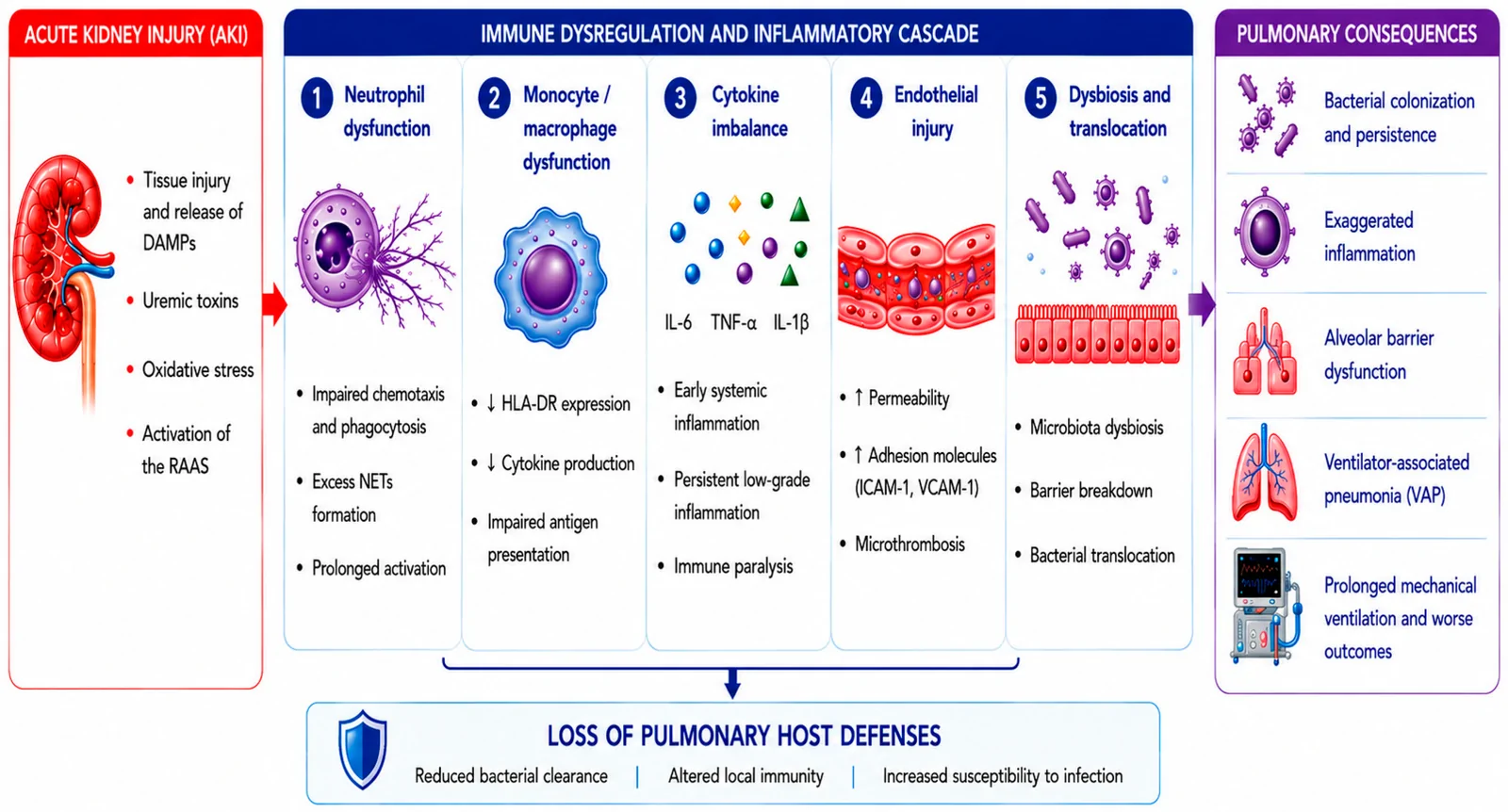
Immunological and inflammatory mechanisms underlying kidney–lung crosstalk. HLA-DR: human leukocyte antigen–DR; IL: interleukin; IL-1β: interleukin-1 beta; IL-6: interleukin-6; NETs: neutrophil extracellular traps; NF-κB: nuclear factor kappa B; TNF-α: tumor necrosis factor alpha; VAP: ventilator-associated pneumonia.

**Figure 3:**
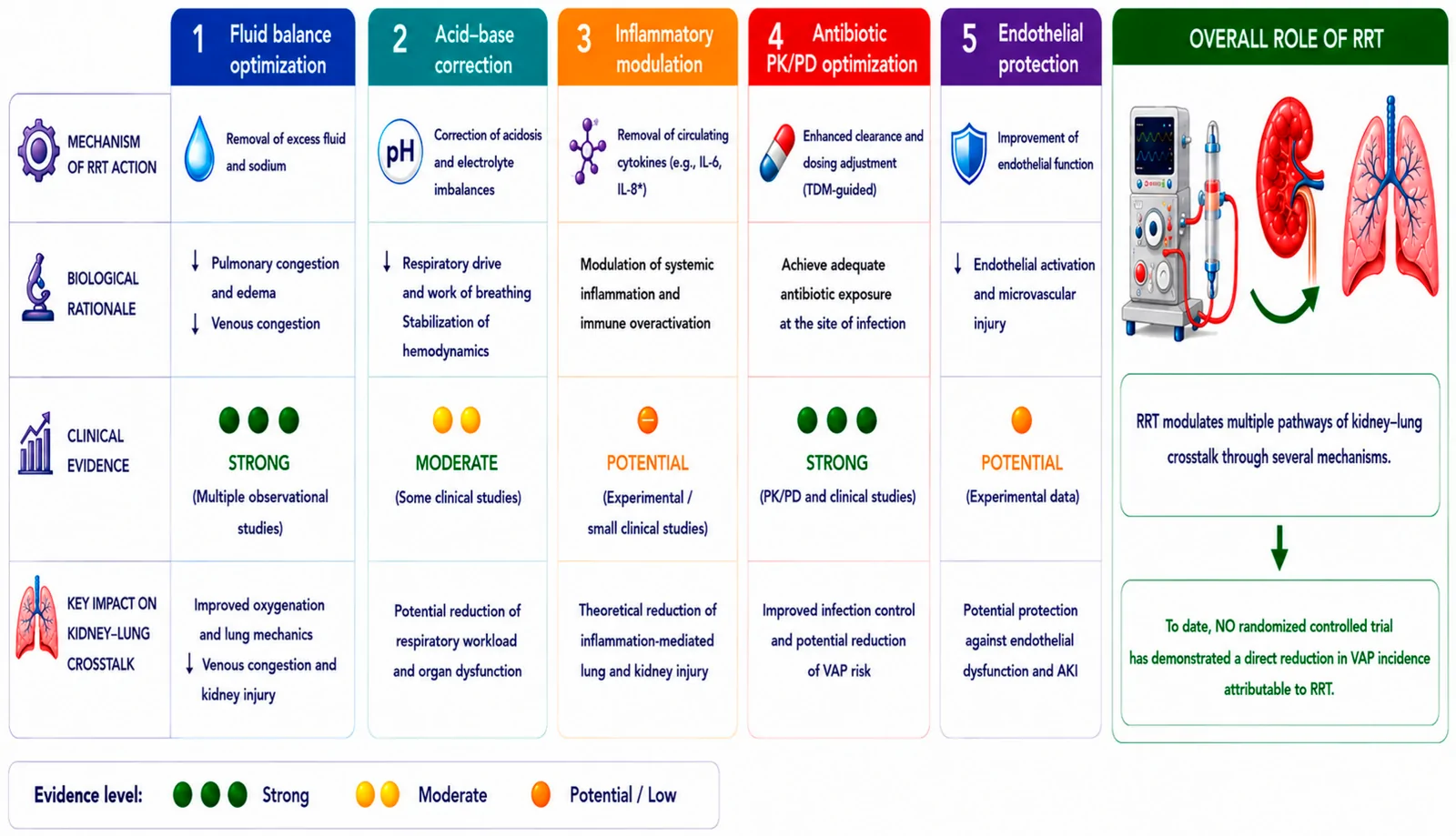
Potential interactions between renal replacement therapy and kidney–lung crosstalk. AKI: acute kidney injury; IL: interleukin; IL-6: interleukin-6; IL-8: interleukin-8; RRT: renal replacement therapy; TDM: therapeutic drug monitoring; VAP: ventilator-associated pneumonia.

**Table 1:** Volemic axis: key studies on fluid overload and respiratory outcomes in the ICU.

Study Year	Design	Population	Exposure/Research Question	Main Results	Main Limitations	Key Message
*A—Fluid overload and mortality in the ICU*						
**SOAP study Payen et al.** 2008 [17]	Prospective cohort	3147 patients ICU 198 European centres	Positive fluid balance in patients with AKI → mortality	Positive fluid balance independently associated with higher mortality in patients with AKI, after adjustment for severity. European reference cohort on the prognostic impact of fluid overload in the ICU.	Observational Residual confounding	Fluid overload + AKI = worse mortality
**FINNAKI Vaara et al.** 2012 [19]	Prospective cohort	2901 patients ICU 17 centres, Finland	Fluid overload at RRT initiation → 90-day mortality	Odds ratio 1.12 per excess litre (*p* < 0.001). Mortality of 60% for fluid overload exceeding 10% of body weight versus 40% in normovolaemic patients.	Observational Residual confounding VAP not directly analysed	Fluid overload = major independent prognostic factor in AKI patients receiving RRT
**Legrand et al.** 2013 [18]	Retrospective cohort	Septic ICU patients Single centre	Central venous pressure → risk of new or persistent AKI	CVP independently associated with AKI risk after adjustment for fluid balance and PEEP level (OR 1.22 [1.08–1.39], *p* = 0.002). Independent role of venous congestion demonstrated beyond fluid balance alone.	Retrospective Single centre	Elevated CVP = independent risk factor for AKI beyond fluid balance
**Silversides et al.** 2018 [14].	Prospective cohort	418 patients Iatrogenic fluid overload Medical ICU	Active de-resuscitation → 60-day mortality	De-resuscitation significantly associated with reduced 60-day mortality (33% vs. 52%, *p* < 0.001 in adjusted analysis) and shorter duration of mechanical ventilation. RRT used as a de-resuscitation tool in 38% of cases.	Observational Residual confounding	Active de-resuscitation associated with reduced mortality and shorter duration of mechanical ventilation
*B—Mechanistic reference data*						
**Prowle, Kirwan & Bellomo** 2014 [11].	Review	Mechanistic synthesis	Fluid management, renal venous congestion and prevention of AKI	Reference mechanistic review on fluid overload and AKI. Formalises the concept of renal venous congestion (reduced renal perfusion pressure gradient) and its consequences on pulmonary compliance and gas exchange. Highest impact factor among mechanistic reviews on this topic.	Narrative review No primary data	Reference mechanistic framework—fluid overload as a bidirectional kidney–lung insult
**Chen et al.** 2016 [12]	Prospective cohort	Ventilated critically ill patients Medical ICU	CVP and peripheral oedema → risk of AKI	Elevated CVP and peripheral oedema significantly associated with higher AKI risk, independently of cardiac output.	Observational Causality not established	Elevated CVP = independent risk factor for AKI regardless of cardiac output
*C—Recent recommendations and synthesis*						
**Ostermann et al. ESICM Guidelines** 2025 [10].	Guidelines	Adult ICU patients De-escalation phase	Active fluid de-escalation in the reversal phase—levels of evidence and recommendations	Active fluid de-escalation recommended during the reversal phase, but available trials do not demonstrate robust renal or respiratory clinical benefit in the general ICU population. Conditional recommendations based on low-to-moderate quality evidence.	Expert consensus Heterogeneous data	Fluid de-escalation recommended but without robust evidence of benefit in the general ICU population

Abbreviations: AKI, acute kidney injury; CVP, central venous pressure; ICU, intensive care unit; PEEP, positive end-expiratory pressure; RRT, renal replacement therapy; VAP, ventilator-associated pneumonia.

**Table 2:** Immuno-inflammatory axis: key studies on cytokine responses, immunoparalysis and their role in susceptibility to nosocomial infection.

Study Year	Design	Population/Model	Research Question/Mechanism Studied	Main Results	Main Limitations	Key Message
*A—Founding experimental studies—direct cytokine-mediated mechanisms*						
**Hoke et al.** 2007 [15].	Experimental	Murine model Bilateral nephrectomy with and without volume control	Does AKI induce acute lung injury through a direct cytokine-mediated mechanism, independently of fluid overload?	First direct experimental demonstration of the AKI-to-lung crosstalk via cytokine-mediated mechanisms. Bilateral nephrectomy induces increased alveolo-capillary permeability, pulmonary neutrophil infiltration and elevated lung cytokines (IL-6, TNF-alpha, CXCL1) in the absence of any volume manipulation. Founding paradigm of the field.	Murine model Limited translational value to humans	Founding study—establishes the paradigm of the direct cytokine-mediated AKI-to-lung crosstalk
**Faubel & Edelstein** 2016 [6].	Review	Experimental and clinical synthesis	Mechanisms and mediators of AKI-induced lung injury—role of circulating cytokines	Reference mechanistic review on AKI-induced lung injury. Synthesises the role of humoral mediators—IL-6, IL-8, TNF-alpha, CXCL1—as the direct molecular interface of the AKI-to-lung crosstalk. Distinguishes direct cytokine-mediated mechanisms from endothelial and metabolic pathways. Highest impact factor among mechanistic reviews on this axis.	Narrative review Data predominantly experimental	Reference mechanistic review—circulating cytokines as the direct molecular interface of the crosstalk
**Mendes et al.** 2024 [32]	Review	2024 mechanistic update	Recent advances in bidirectional lung-kidney crosstalk mechanisms	2024 update of bidirectional crosstalk mechanisms. Integrates the emerging role of extracellular vesicles and connexins as novel vectors of inter-organ pro-inflammatory signalling. Confirms VILI as a major mechanism of the lung-to-kidney crosstalk. Notes that experimentally identified therapeutic targets have not yet demonstrated clinical efficacy.	Narrative review No meta-analysis	Most recent mechanistic reference—extracellular vesicles and connexins as novel actors
*B—Clinical data—AKI-induced immunoparalysis and susceptibility to nosocomial infection*						
**Hoste et al.** 2018 [47]	Global cohort	13.3 million cases estimated per year International cohort analysis	Global epidemiology of AKI—association with nosocomial infections and ICU mortality	13.3 million AKI cases estimated annually worldwide. In the ICU, AKI is associated with increased nosocomial infections and a 3- to 5-fold higher 90-day mortality depending on KDIGO stage.	Heterogeneity of definitions across cohorts Selection bias	Global epidemiological reference—AKI associated with nosocomial infections and 3- to 5-fold higher mortality
**Zarbock et al. ADQI 28 Consensus** 2023 [48]	Consensus	28 international experts Sepsis-associated AKI	International consensus on sepsis-associated AKI—mechanisms, biomarkers, therapeutic implications	Most recent reference consensus on septic AKI. Explicitly acknowledges AKI-induced immune dysfunction—reduced monocyte HLA-DR expression, neutrophil and macrophage dysfunction—as a determinant of susceptibility to nosocomial infections. Highlights the difficulty of quantifying its isolated clinical impact.	Expert consensus No primary data	Recommendatory framework—AKI-induced immune dysfunction leading to nosocomial infections recognised internationally
*C—Supplementary experimental data—neutrophils and immunoparalysis*						
**Mulay & Anders** 2020 [33].	Review	Mechanistic synthesis Neutrophils and extracellular traps	Role of neutrophils and deoxyribonucleoprotein extracellular traps in immune responses in health and disease	Synthesis of the role of deoxyribonucleoprotein extracellular traps (NETs) in direct tissue injury and pulmonary immunoparalysis. AKI-activated neutrophils release NETs into the lung parenchyma, simultaneously contributing to alveolar injury and functional exhaustion of local defences.	Mechanistic review Limited direct clinical translation	Key mechanism—NETs: direct tissue injury and pulmonary immunoparalysis
*D—Limitations of direct clinical evidence for the cytokine axis*						
**Manrique-Caballero et al.** 2021 [31].	Review	AKI-sepsis synthesis	Sepsis-associated AKI—pathophysiological mechanisms and limitations of clinical evidence for inflammatory pathways	Reference review on septic AKI mechanisms. Highlights that direct clinical demonstration of the cytokine pathway’s impact remains limited, as the multiplicity of inflammatory sources in the ICU prevents isolation of AKI’s specific contribution. The failure of anti-cytokine trials in sepsis illustrates the gap between experimental plausibility and clinical translation.	Narrative review	Gap between experimental plausibility and clinical demonstration—failure of anti-cytokine trials in sepsis
**Sullivan, Liu & Matthay** 2025 [7].	Review	2025 mechanistic synthesis—Lung-kidney crosstalk	Mechanisms of lung-kidney crosstalk in acute injury—2025 update	Most recent review (2025) on inter-organ crosstalk in critical illness. Details ARDS-AKI interactions, integrates advances on extracellular vesicles and inter-organ epigenetic interactions. Highlights bidirectionality of injury and identifies potential therapeutic targets—while noting that clinical evidence for their efficacy remains to be established.	Narrative review No meta-analysis	Most recent conceptual reference—important signal of up-to-date knowledge for reviewers

Abbreviations: AKI, acute kidney injury; ARDS, acute respiratory distress syndrome; CXCL1, C-X-C motif chemokine ligand 1; HLA-DR, human leukocyte antigen-DR; ICU, intensive care unit; IL, interleukin; KDIGO, Kidney Disease: Improving Global Outcomes; NETs, neutrophil extracellular traps; TNF-α, tumor necrosis factor alpha; VILI, ventilator-induced lung injury.

**Table 3:** Experimental and Clinical Evidence Supporting the Role of Renal Replacement Therapy (RRT) in Kidney–Lung Crosstalk.

Pathway	Experimental Evidence	Clinical Evidence	Interventional Evidence	Key References
Fluid overload/congestion	+++	+++	++	[3,11,12,14,17,18,19,25,26,27,28,74,89,104,105]
Immune-inflammatory	+++	++	+	[6,15,32,48]
Endothelial dysfunction	++	+	0	[3,6,49,50,51]
Uremic toxins	++	0	0	[6,59,60,61]
Mechanical ventilation	+++	++	++	[62,63,64,65,71]
VILI	+++	++	++	[32,66,69,71]
Hypoxemia/hypercapnia	++	+	0	[72,73]
Extracorporeal cytokine removal	++	+	0	[88,92,93,94]
Antibiotic PK optimization	+	+++	+++	[95,96,97,98,106]

Evidence strength is presented qualitatively: **0**, no or insufficient evidence; **+**, limited evidence; **++**, moderate evidence; **+++**, strong and/or consistent evidence. This grading is intended as a narrative synthesis of the available literature and does not represent a formal evidence-grading system. **Abbreviations:** RRT, renal replacement therapy; VILI, ventilator-induced lung injury; PK, pharmacokinetics.

## Data Availability

No new data were created or analyzed in this study. Data sharing is not applicable to this article.

## Supplementary Materials

The following supporting information can be downloaded at: https://www.mdpi.com/article/10.3390/jcm15197570/s1, Table S1: Fluid resuscitation strategies and renal outcomes in the ICU; Table S2: Immunomodulatory agents and renal outcomes in the ICU: corticosteroids, anti-IL-6 and JAK inhibitors in CAP, ARDS and COVID-19; Table S3: Sodium bicarbonate in the ICU (BICAR-ICU/BICARICU-2): impact on respiratory function and the kidney-lung crosstalk; Table S4: Timing of RRT initiation: impact on respiratory function. Figure S1: Flow-chart of the study selection.

## Abbreviations

AKI: Acute Kidney Injury; ARDS: Acute Respiratory Distress Syndrome; AUC: Area Under the Curve; CAPE COD: Community-Acquired Pneumonia: Evaluation of Corticosteroids; CPIS: Clinical Pulmonary Infection Score; CRRT: Continuous Renal Replacement Therapy; CVP: Central Venous Pressure; ERS: European Respiratory Society; ESICM: European Society of Intensive Care Medicine; ESCMID: European Society of Clinical Microbiology and Infectious Diseases; FO: Fluid Overload; ICU: Intensive Care Unit; KDIGO: Kidney Disease: Improving Global Outcomes; MODS: Multiple Organ Dysfunction Syndrome; MV: Mechanical Ventilation; NETs: Neutrophil Extracellular Traps; NHSN: National Healthcare Safety Network; PEEP: Positive End-Expiratory Pressure; RIFLE: Risk, Injury, Failure, Loss of Kidney Function, End-Stage Kidney Disease; RRT: Renal Replacement Therapy; RV: Right Ventricle; SCCM: Society of Critical Care Medicine; VAP: Ventilator-Associated Pneumonia; VExUS: Venous Excess Ultrasound Score; VILI: Ventilator-Induced Lung Injury.

## References

[1] Jarczak D., Kluge S., Nierhaus A. (2021). Sepsis—Pathophysiology and Therapeutic Concepts. Front. Med..

[2] Srdić T., Đurašević S., Lakić I., Ružičić A., Vujović P., Jevđović T., Dakić T., Đorđević J., Tosti T., Glumac S. (2024). From Molecular Mechanisms to Clinical Therapy: Understanding Sepsis-Induced Multiple Organ Dysfunction. Int. J. Mol. Sci..

[3] Husain-Syed F., Slutsky A.S., Ronco C. (2016). Lung–Kidney Cross-Talk in the Critically Ill Patient. Am. J. Respir. Crit. Care Med..

[4] Ronco C., Bellomo R., Kellum J.A. (2019). Acute Kidney Injury. Lancet.

[5] Doi K., Rabb H. (2016). Impact of Acute Kidney Injury on Distant Organ Function: Recent Findings and Potential Therapeutic Targets. Kidney Int..

[6] Faubel S., Edelstein C.L. (2016). Mechanisms and Mediators of Lung Injury after Acute Kidney Injury. Nat. Rev. Nephrol..

[7] Sullivan K.M., Liu K.D., Matthay M.A. (2025). Organ Crosstalk During Injury: Mechanisms of Lung–Kidney Interaction in Critical Illness. Compr. Physiol..

[8] Borgatta B., Rello J. (2014). How to Approach and Treat VAP in ICU Patients. BMC Infect. Dis..

[9] Torres A., Niederman M.S., Chastre J., Ewig S., Fernandez-Vandellos P., Hanberger H., Kollef M., Bassi G.L., Luna C.M., Martin-Loeches I. (2017). International ERS/ESICM/ESCMID/ALAT Guidelines for the Management of Hospital-Acquired Pneumonia and Ventilator-Associated Pneumonia: Guidelines for the Management of Hospital-Acquired Pneumonia (HAP)/Ventilator-Associated Pneumonia (VAP) of the European Respiratory Society (ERS), European Society of Intensive Care Medicine (ESICM), European Society of Clinical Microbiology and Infectious Diseases (ESCMID) and Asociación Latinoamericana Del Tórax (ALAT). Eur. Respir. J..

[10] Ostermann M., Lumlertgul N., Jeong R., See E., Joannidis M., James M. (2025). Acute Kidney Injury. Lancet.

[11] Prowle J.R., Kirwan C.J., Bellomo R. (2014). Fluid Management for the Prevention and Attenuation of Acute Kidney Injury. Nat. Rev. Nephrol..

[12] Chen K.P., Cavender S., Lee J., Feng M., Mark R.G., Celi L.A., Mukamal K.J., Danziger J. (2016). Peripheral Edema, Central Venous Pressure, and Risk of AKI in Critical Illness. Clin. J. Am. Soc. Nephrol..

[13] Wang H., He H. (2020). Expiratory Flow Limitation Developed in ICU Patients: Relationship of Fluid Overload, Respiratory Mechanics, and Outcome. Crit. Care.

[14] Silversides J.A., Fitzgerald E., Manickavasagam U.S., Lapinsky S.E., Nisenbaum R., Hemmings N., Nutt C., Trinder T.J., Pogson D.G., Fan E. (2018). Deresuscitation of Patients With Iatrogenic Fluid Overload Is Associated With Reduced Mortality in Critical Illness*. Crit. Care Med..

[15] Hoke T.S., Douglas I.S., Klein C.L., He Z., Fang W., Thurman J.M., Tao Y., Dursun B., Voelkel N.F., Edelstein C.L. (2007). Acute Renal Failure after Bilateral Nephrectomy Is Associated with Cytokine-Mediated Pulmonary Injury. J. Am. Soc. Nephrol..

[16] Yang B., Yang C., Wang Y. (2022). Editorial: Innate Immunity in Kidney Injury, Repair and Fibrosis. Front. Immunol..

[17] Payen D., De Pont A.C., Sakr Y., Spies C., Reinhart K., Vincent J.L. (2008). A Positive Fluid Balance Is Associated with a Worse Outcome in Patients with Acute Renal Failure. Crit. Care.

[18] Legrand M., Dupuis C., Simon C., Gayat E., Mateo J., Lukaszewicz A.-C., Payen D. (2013). Association between Systemic Hemodynamics and Septic Acute Kidney Injury in Critically Ill Patients: A Retrospective Observational Study. Crit. Care.

[19] Vaara S.T., Korhonen A.-M., Kaukonen K.-M., Nisula S., Inkinen O., Hoppu S., Laurila J.J., Mildh L., Reinikainen M., Lund V. (2012). Fluid Overload Is Associated with an Increased Risk for 90-Day Mortality in Critically Ill Patients with Renal Replacement Therapy: Data from the Prospective FINNAKI Study. Crit. Care.

[20] Hayashi Y., Shimazui T., Tomita K., Shimada T., Miura R.E., Nakada T. (2023). Associations between Fluid Overload and Outcomes in Critically Ill Patients with Acute Kidney Injury: A Retrospective Observational Study. Sci. Rep..

[21] Husain-Syed F., Gröne H.-J., Assmus B., Bauer P., Gall H., Seeger W., Ghofrani A., Ronco C., Birk H.-W. (2021). Congestive Nephropathy: A Neglected Entity? Proposal for Diagnostic Criteria and Future Perspectives. ESC Heart Fail..

[22] Ranjit S., Rola P., Natraj R., Bhaskaran A.K., Haycock K., Siuba M.T. (2024). Venous Congestion Assessed by Venous Excess Ultrasound (VExUS) and Acute Kidney Injury in Children with Right Ventricular Dysfunction. Indian J. Crit. Care Med..

[23] Vieillard-Baron A., Naeije R., Haddad F., Bogaard H.J., Bull T.M., Fletcher N., Lahm T., Magder S., Orde S., Schmidt G. (2018). Diagnostic Workup, Etiologies and Management of Acute Right Ventricle Failure: A State-of-the-Art Paper. Intensive Care Med..

[24] Beaubien-Souligny W., Rola P., Haycock K., Bouchard J., Lamarche Y., Spiegel R., Denault A.Y. (2020). Quantifying Systemic Congestion with Point-Of-Care Ultrasound: Development of the Venous Excess Ultrasound Grading System. Ultrasound J..

[25] Wiedemann H.P., Wheeler A.P., Bernard G.R., Thompson B.T., Hayden D., de Boisblanc B., Connors A.F., Hite R.D., Harabin A.L., National Heart, Lung, and Blood Institute Acute Respiratory Distress Syndrome (ARDS) Clinical Trials Network (2006). Comparison of Two Fluid-Management Strategies in Acute Lung Injury. N. Engl. J. Med..

[26] Meyhoff T.S., Hjortrup P.B., Wetterslev J., Sivapalan P., Laake J.H., Cronhjort M., Jakob S.M., Cecconi M., Nalos M., Ostermann M. (2022). Restriction of Intravenous Fluid in ICU Patients with Septic Shock. N. Engl. J. Med..

[27] Shapiro N.I., Douglas I.S., Brower R.G., Brown S.M., Exline M.C., Ginde A.A., Gong M.N., Grissom C.K., Hayden D., National Heart, Lung, and Blood Institute Prevention and Early Treatment of Acute Lung Injury Clinical Trials Network (2023). Early Restrictive or Liberal Fluid Management for Sepsis-Induced Hypotension. N. Engl. J. Med..

[28] Vaara S.T., Ostermann M., Bitker L., Schneider A., Poli E., Hoste E., Fierens J., Joannidis M., Zarbock A., Van Haren F. (2021). Restrictive Fluid Management versus Usual Care in Acute Kidney Injury (REVERSE-AKI): A Pilot Randomized Controlled Feasibility Trial. Intensive Care Med..

[29] Ostermann M., AlShamsi F., Raventos A.A., Cecconi M., Ichai C., Jones C.E., Malbrain M.L.N.G., Monnet X., Nalos M., Peng Z. (2025). European Society of Intensive Care Medicine Clinical Practice Guideline on Fluid Therapy in Adult Critically Ill Patients: Part 3—Fluid Removal at de-Escalation Phase. Intensive Care Med..

[30] Malbrain M.L.N.G., Van Regenmortel N., Saugel B., De Tavernier B., Van Gaal P.-J., Joannes-Boyau O., Teboul J.-L., Rice T.W., Mythen M., Monnet X. (2018). Principles of Fluid Management and Stewardship in Septic Shock: It Is Time to Consider the Four D’s and the Four Phases of Fluid Therapy. Ann. Intensive Care.

[31] Manrique-Caballero C.L., Del Rio-Pertuz G., Gomez H. (2021). Sepsis-Associated Acute Kidney Injury. Crit. Care Clin..

[32] Mendes R.D.S., Silva P.L., Robba C., Battaglini D., Lopes-Pacheco M., Caruso-Neves C., Rocco P.R.M. (2024). Advancements in Understanding the Mechanisms of Lung–Kidney Crosstalk. Intensive Care Med. Exp..

[33] Mulay S.R., Anders H.-J. (2020). Neutrophils and Neutrophil Extracellular Traps Regulate Immune Responses in Health and Disease. Cells.

[34] Saavedra-Torres J.S., Pinzón-Fernández M.V., Nati-Castillo H.A., Cadena Correa V., Lopez Molina L.C., Gaitán J.E., Tenorio-Castro D., Lucero Guanga D.A., Arias-Intriago M., Tello-De-la-Torre A. (2025). Immunodynamic Disruption in Sepsis: Mechanisms and Strategies for Personalized Immunomodulation. Biomedicines.

[35] Ruault A., Philipponnet C., Brailova M., Gamara F., Sapin V., Evrard B., Calvet L., Bonnet B., Adda M., Souweine B. (2025). Biomarkers and Early-Onset Acute Kidney Injury in Critically Ill COVID-19 Patients, a Prospective Monocentric Observational Study. BMC Nephrol..

[36] Meduri G.U., Shih M.-C., Bridges L., Martin T.J., El-Solh A., Seam N., Davis-Karim A., Umberger R., Anzueto A., Sriram P. (2022). Low-Dose Methylprednisolone Treatment in Critically Ill Patients with Severe Community-Acquired Pneumonia. Intensive Care Med..

[37] Torres A., Sibila O., Ferrer M., Polverino E., Menendez R., Mensa J., Gabarrús A., Sellarés J., Restrepo M.I., Anzueto A. (2015). Effect of Corticosteroids on Treatment Failure among Hospitalized Patients with Severe Community-Acquired Pneumonia and High Inflammatory Response: A Randomized Clinical Trial. JAMA.

[38] Meduri G.U., Golden E., Freire A.X., Taylor E., Zaman M., Carson S.J., Gibson M., Umberger R. (2007). Methylprednisolone Infusion in Early Severe ARDS: Results of a Randomized Controlled Trial. Chest.

[39] (2022). RECOVERY Collaborative Group Baricitinib in Patients Admitted to Hospital with COVID-19 (RECOVERY): A Randomised, Controlled, Open-Label, Platform Trial and Updated Meta-Analysis. Lancet.

[40] Gaudry S., Hajage D., Martin-Lefevre L., Lebbah S., Louis G., Moschietto S., Titeca-Beauport D., Combe B.L., Pons B., de Prost N. (2021). Comparison of Two Delayed Strategies for Renal Replacement Therapy Initiation for Severe Acute Kidney Injury (AKIKI 2): A Multicentre, Open-Label, Randomised, Controlled Trial. Lancet.

[41] The RECOVERY Collaborative Group (2021). Dexamethasone in Hospitalized Patients with Covid-19. N. Engl. J. Med..

[42] Abani O., Abbas A., Abbas F., Abbas M., Abbasi S., Abbass H., Abbott A., Abdallah N., Abdelaziz A., Abdelfattah M. (2021). Tocilizumab in Patients Admitted to Hospital with COVID-19 (RECOVERY): A Randomised, Controlled, Open-Label, Platform Trial. Lancet.

[43] Waring R. (2006). Efficacy and Safety of Corticosteroids for Persistent Acute Respiratory Distress Syndrome. N. Engl. J. Med..

[44] Dequin P.-F., Meziani F., Quenot J.-P., Kamel T., Ricard J.-D., Badie J., Reignier J., Heming N., Plantefève G., Souweine B. (2023). Hydrocortisone in Severe Community-Acquired Pneumonia. N. Engl. J. Med..

[45] Bolisetty S., Agarwal A. (2009). Neutrophils in Acute Kidney Injury: Not Neutral Any More. Kidney Int..

[46] Sharfuddin A.A., Molitoris B.A. (2011). Pathophysiology of Ischemic Acute Kidney Injury. Nat. Rev. Nephrol..

[47] Hoste E.A.J., Kellum J.A., Selby N.M., Zarbock A., Palevsky P.M., Bagshaw S.M., Goldstein S.L., Cerdá J., Chawla L.S. (2018). Global Epidemiology and Outcomes of Acute Kidney Injury. Nat. Rev. Nephrol..

[48] Zarbock A., Nadim M.K., Pickkers P., Gomez H., Bell S., Joannidis M., Kashani K., Koyner J.L., Pannu N., Meersch M. (2023). Sepsis-Associated Acute Kidney Injury: Consensus Report of the 28th Acute Disease Quality Initiative Workgroup. Nat. Rev. Nephrol..

[49] Dellepiane S., Marengo M., Cantaluppi V. (2016). Detrimental Cross-Talk between Sepsis and Acute Kidney Injury: New Pathogenic Mechanisms, Early Biomarkers and Targeted Therapies. Crit. Care.

[50] Uchimido R., Schmidt E.P., Shapiro N.I. (2019). The Glycocalyx: A Novel Diagnostic and Therapeutic Target in Sepsis. Crit. Care.

[51] Patterson E.K., Cepinskas G., Fraser D.D. (2022). Endothelial Glycocalyx Degradation in Critical Illness and Injury. Front. Med..

[52] Liu H., Dong J., Xu C., Ni Y., Ye Z., Sun Z., Fan H., Chen Y. (2025). Acute Lung Injury: Pathogenesis and Treatment. J. Transl. Med..

[53] Iba T., Maier C.L., Helms J., Ferrer R., Thachil J., Levy J.H. (2024). Managing Sepsis and Septic Shock in an Endothelial Glycocalyx-Friendly Way: From the Viewpoint of Surviving Sepsis Campaign Guidelines. Ann. Intensive Care.

[54] Kraut J.A., Madias N.E. (2010). Metabolic Acidosis: Pathophysiology, Diagnosis and Management. Nat. Rev. Nephrol..

[55] Jaber S., Paugam C., Futier E., Lefrant J.-Y., Lasocki S., Lescot T., Pottecher J., Demoule A., Ferrandière M., Asehnoune K. (2018). Sodium Bicarbonate Therapy for Patients with Severe Metabolic Acidaemia in the Intensive Care Unit (BICAR-ICU): A Multicentre, Open-Label, Randomised Controlled, Phase 3 Trial. Lancet.

[56] Jung B., Jabaudon M., De Jong A., Bitker L., Audard J., Klouche K., Sarton B., Guitton C., Lasocki S., Rieu B. (2025). Sodium Bicarbonate for Severe Metabolic Acidemia and Acute Kidney Injury: The BICARICU-2 Randomized Clinical Trial. JAMA.

[57] Semler M.W., Self W.H., Wanderer J.P., Ehrenfeld J.M., Wang L., Byrne D.W., Stollings J.L., Kumar A.B., Hughes C.G., Hernandez A. (2018). Balanced Crystalloids versus Saline in Critically Ill Adults. N. Engl. J. Med..

[58] Self W.H., Semler M.W., Wanderer J.P., Wang L., Byrne D.W., Collins S.P., Slovis C.M., Lindsell C.J., Ehrenfeld J.M., Siew E.D. (2018). Balanced Crystalloids versus Saline in Noncritically Ill Adults. N. Engl. J. Med..

[59] Pindjakova J., Griffin M.D. (2011). Defective Neutrophil Rolling and Transmigration in Acute Uremia. Kidney Int..

[60] Cohen G., Vanholder R. (2021). Special Issue: Immune Dysfunction in Uremia. Toxins.

[61] Kaysi S., Taghavi M., El Mourabi A., Antoine M.-H., De Prez E., Nortier J. (2026). Uremic Toxins and the Lung Alveolar Capillary Barrier: A Narrative Review. Toxins.

[62] Kumar A., Epler K., DeWolf S., Barnes L., Hepokoski M. (2024). Bidirectional Pressure: A Mini Review of Ventilator-Lung-Kidney Interactions. Front. Physiol..

[63] Benites M.H., Suarez-Sipmann F., Kattan E., Cruces P., Retamal J. (2025). Ventilation-Induced Acute Kidney Injury in Acute Respiratory Failure: Do PEEP Levels Matter?. Crit. Care.

[64] Poirot L., Wangueu L.T., Breteau I., Petit M., Schmidt M., Bavozet F., Mercat A., Asfar P., Beloncle F., COVID ICU Group on behalf of the REVA Network and the COVID-ICU Investigators (2025). PEEP-AKI-COVID ICU: Effect of Positive End-Expiratory Pressure on Acute Kidney Injury Development in Patients with COVID-19-Associated Acute Respiratory Distress Syndrome: An Ancillary Analysis of the COVID-ICU Study. J. Intensive Care.

[65] Meersch-Dini M., Abu-Tair M., Bayer M., Brinkmann A., Bellmann R., Brunkhorst F., Custodis F., Czock D., Frey O., Galle J. (2026). Multidisciplinary Guidelines on Renal Replacement Therapy in Intensive Care Medicine. Crit. Care.

[66] Curley G.F., Laffey J.G., Zhang H., Slutsky A.S. (2016). Biotrauma and Ventilator-Induced Lung Injury. Chest.

[67] Sweeney R.M., McAuley D.F. (2016). Acute Respiratory Distress Syndrome. Lancet.

[68] Li Z.-L., Li X.-Y., Zhou Y., Wang B., Lv L.-L., Liu B.-C. (2024). Renal Tubular Epithelial Cells Response to Injury in Acute Kidney Injury. eBioMedicine.

[69] Qiao X., Yin J., Zheng Z., Li L., Feng X. (2024). Endothelial Cell Dynamics in Sepsis-Induced Acute Lung Injury and Acute Respiratory Distress Syndrome: Pathogenesis and Therapeutic Implications. Cell Commun. Signal..

[70] Antonucci E., Garcia B., Chen D., Matthay M.A., Liu K.D., Legrand M. (2024). Incidence of Acute Kidney Injury and Attributive Mortality in Acute Respiratory Distress Syndrome Randomized Trials. Intensive Care Med..

[71] Fan E., Del Sorbo L., Goligher E.C., Hodgson C.L., Munshi L., Walkey A.J., Adhikari N.K.J., Amato M.B.P., Branson R., Brower R.G. (2017). An Official American Thoracic Society/European Society of Intensive Care Medicine/Society of Critical Care Medicine Clinical Practice Guideline: Mechanical Ventilation in Adult Patients with Acute Respiratory Distress Syndrome. Am. J. Respir. Crit. Care Med..

[72] Marchiset A., Jamme M. (2022). When the Renal (Function) Begins to Fall: A Mini-Review of Acute Kidney Injury Related to Acute Respiratory Distress Syndrome in Critically Ill Patients. Front. Nephrol..

[73] De Rosa S., Lassola S., Taccone F.S., Battaglini D. (2025). Chronic Lung Diseases and Kidney Disease: Pathophysiology and Management. Nephrol. Dial. Transplant..

[74] Legrand M., Khanna A.K., Ostermann M., Kotani Y., Ferrer R., Girardis M., Leone M., DePascale G., Pickkers P., Tissieres P. (2024). The Renin–Angiotensin–Aldosterone-System in Sepsis and Its Clinical Modulation with Exogenous Angiotensin II. Crit. Care.

[75] Singbartl K., Bishop J.V., Wen X., Murugan R., Chandra S., Filippi M.-D., Kellum J.A. (2011). Differential Effects of Kidney–Lung Cross-Talk during Acute Kidney Injury and Bacterial Pneumonia. Kidney Int..

[76] Papazian L., Klompas M., Luyt C.-E. (2020). Ventilator-Associated Pneumonia in Adults: A Narrative Review. Intensive Care Med..

[77] Matthay M.A., Arabi Y., Arroliga A.C., Bernard G., Bersten A.D., Brochard L.J., Calfee C.S., Combes A., Daniel B.M., Ferguson N.D. (2024). A New Global Definition of Acute Respiratory Distress Syndrome. Am. J. Respir. Crit. Care Med..

[78] Levi T.M., Souza S.P.D., Magalhães J.G.D., Carvalho M.S.D., Cunha A.L.B., Dantas J.G.A.D.O., Cruz M.G., Guimarães Y.L.M., Cruz C.M.S. (2013). Comparison of the RIFLE, AKIN and KDIGO Criteria to Predict Mortality in Critically Ill Patients. Rev. Bras. Ter. Intensiv..

[79] Zeng X., McMahon G.M., Brunelli S.M., Bates D.W., Waikar S.S. (2014). Incidence, Outcomes, and Comparisons across Definitions of AKI in Hospitalized Individuals. Clin. J. Am. Soc. Nephrol..

[80] Wolkewitz M., Mandel M., Palomar-Martinez M., Alvarez-Lerma F., Olaechea-Astigarraga P., Schumacher M. (2018). Methodological Challenges in Using Point-Prevalence versus Cohort Data in Risk Factor Analyses of Nosocomial Infections. Ann. Epidemiol..

[81] Hazard D., Grodd M., Walzer D., Biever P., Rieg S., Wolkewitz M. (2025). How to Avoid Time-Related Types of Bias in the Analysis of Clinical Infectious Diseases: Demonstration and Methods. Clin. Microbiol. Infect..

[82] Suissa S. (2008). Immortal Time Bias in Pharmacoepidemiology. Am. J. Epidemiol..

[83] Kyriacou D.N., Lewis R.J. (2016). Confounding by Indication in Clinical Research. JAMA.

[84] Peerapornratana S., Manrique-Caballero C.L., Gómez H., Kellum J.A. (2019). Acute Kidney Injury from Sepsis: Current Concepts, Epidemiology, Pathophysiology, Prevention and Treatment. Kidney Int..

[85] Ince C., Mayeux P.R., Nguyen T., Gomez H., Kellum J.A., Ospina-Tascón G.A., Hernandez G., Murray P., De Backer D. (2016). The Endothelium in Sepsis. Shock.

[86] De Andrade L.V., Vasconcelos B., Khouri R., De Souza C.D.F., Armstrong A.D.C., Do Carmo R.F. (2025). Impact of Complement System Proteins on the Clinical Progression of Hospitalized Patients with COVID-19. BMC Infect. Dis..

[87] Mingeot-Leclercq M.-P., Tulkens P.M. (1999). Aminoglycosides: Nephrotoxicity. Antimicrob. Agents Chemother..

[88] Bottari G., Ranieri V.M., Ince C., Pesenti A., Aucella F., Scandroglio A.M., Ronco C., Vincent J.-L. (2024). Use of Extracorporeal Blood Purification Therapies in Sepsis: The Current Paradigm, Available Evidence, and Future Perspectives. Crit. Care.

[89] Bitker L., Dupuis C., Pradat P., Deniel G., Klouche K., Mezidi M., Chauvelot L., Yonis H., Baboi L., Illinger J. (2024). Fluid Balance Neutralization Secured by Hemodynamic Monitoring versus Protocolized Standard of Care in Patients with Acute Circulatory Failure Requiring Continuous Renal Replacement Therapy: Results of the GO NEUTRAL Randomized Controlled Trial. Intensive Care Med..

[90] Kimmoun A., Novy E., Auchet T., Ducrocq N., Levy B. (2015). Hemodynamic Consequences of Severe Lactic Acidosis in Shock States: From Bench to Bedside. Crit. Care.

[91] Ronco C., Bagshaw S.M., Bellomo R., Clark W.R., Husain-Syed F., Kellum J.A., Ricci Z., Rimmelé T., Reis T., Ostermann M. (2021). Extracorporeal Blood Purification and Organ Support in the Critically Ill Patient during COVID-19 Pandemic: Expert Review and Recommendation. Blood Purif..

[92] Lumlertgul N., Hall A., Camporota L., Crichton S., Ostermann M. (2021). Clearance of Inflammatory Cytokines in Patients with Septic Acute Kidney Injury during Renal Replacement Therapy Using the EMiC2 Filter (Clic-AKI Study). Crit. Care.

[93] Steindl D., Schroeder T., Krannich A., Nee J. (2025). Hemoadsorption in the Management of Septic Shock: A Systematic Review and Meta-Analysis. J. Clin. Med..

[94] Waalders N.J.B., Kox M., Pickkers P. (2025). Haemoadsorption to Remove Inflammatory Mediators in Sepsis: Past, Present, and Future. Intensive Care Med. Exp..

[95] Lewis S.J., Mueller B.A. (2024). Antibiotic Dosing Recommendations in Critically Ill Patients Receiving New Innovative Kidney Replacement Therapy. BMC Nephrol..

[96] Roger C. (2024). Understanding Antimicrobial Pharmacokinetics in Critically Ill Patients to Optimize Antimicrobial Therapy: A Narrative Review. J. Intensive Med..

[97] Roberts J.A., Abdul-Aziz M.H., Lipman J., Mouton J.W., Vinks A.A., Felton T.W., Hope W.W., Farkas A., Neely M.N., Schentag J.J. (2014). Individualised Antibiotic Dosing for Patients Who Are Critically Ill: Challenges and Potential Solutions. Lancet Infect. Dis..

[98] Rybak M.J., Le J., Lodise T.P., Levine D.P., Bradley J.S., Liu C., Mueller B.A., Pai M.P., Wong-Beringer A., Rotschafer J.C. (2020). Therapeutic Monitoring of Vancomycin for Serious Methicillin-Resistant *Staphylococcus aureus* Infections: A Revised Consensus Guideline and Review by the American Society of Health-System Pharmacists, the Infectious Diseases Society of America, the Pediatric Infectious Diseases Society, and the Society of Infectious Diseases Pharmacists. Am. J. Health Syst. Pharm..

[99] Zarbock A., Kellum J.A., Schmidt C., Van Aken H., Wempe C., Pavenstädt H., Boanta A., Gerß J., Meersch M. (2016). Effect of Early vs Delayed Initiation of Renal Replacement Therapy on Mortality in Critically Ill Patients With Acute Kidney Injury: The ELAIN Randomized Clinical Trial. JAMA.

[100] Gaudry S., Hajage D., Schortgen F., Martin-Lefevre L., Pons B., Boulet E., Boyer A., Chevrel G., Lerolle N., Carpentier D. (2016). Initiation Strategies for Renal-Replacement Therapy in the Intensive Care Unit. N. Engl. J. Med..

[101] Barbar S.D., Clere-Jehl R., Bourredjem A., Hernu R., Montini F., Bruyère R., Lebert C., Bohé J., Badie J., Eraldi J.-P. (2018). Timing of Renal-Replacement Therapy in Patients with Acute Kidney Injury and Sepsis. N. Engl. J. Med..

[102] The STARRT-AKI Investigators (2020). Timing of Initiation of Renal-Replacement Therapy in Acute Kidney Injury. N. Engl. J. Med..

[103] Jourdain M., Chatti I.G., Housni B., Jaquet P., Jezequel M., Kane O., La Combe B., Landais M., Marzouk M., De Montmollin E. (2025). Renal Replacement Therapy in an Intensive Care Unit: Guidelines from the SRLF-GFRUP Consensus Conference. Ann. Intensive Care.

[104] Corl K.A., Prodromou M., Merchant R.C., Gareen I., Marks S., Banerjee D., Amass T., Abbasi A., Delcompare C., Palmisciano A. (2019). The Restrictive IV Fluid Trial in Severe Sepsis and Septic Shock (RIFTS): A Randomized Pilot Study. Crit. Care Med..

[105] Naorungroj T., Prajantasen U., Sanla-ead T., Viarasilpa T., Tongyoo S. (2025). Restrictive Fluid Management with Early De-Escalation versus Usual Care in Critically Ill Patients (Reduce Trial): A Feasibility Randomized Controlled Trial. Crit. Care.

[106] Guilhaumou R., Benaboud S., Bennis Y., Dahyot-Fizelier C., Dailly E., Gandia P., Goutelle S., Lefeuvre S., Mongardon N., Roger C. (2019). Optimization of the Treatment with Beta-Lactam Antibiotics in Critically Ill Patients—Guidelines from the French Society of Pharmacology and Therapeutics (Société Française de Pharmacologie et Thérapeutique—SFPT) and the French Society of Anaesthesia and Intensive Care Medicine (Société Française d’Anesthésie et Réanimation—SFAR). Crit. Care.

[107] Wang M., Zhu B., Jiang L., Luo X., Wang N., Zhu Y., Xi X., Beijing Acute Kidney Injury Trial (BAKIT) Workgroup (2022). Association between Latent Trajectories of Fluid Balance and Clinical Outcomes in Critically Ill Patients with Acute Kidney Injury: A Prospective Multicenter Observational Study. Kidney Dis..

[108] Klompas M., Branson R., Cawcutt K., Crist M., Eichenwald E.C., Greene L.R., Lee G., Maragakis L.L., Powell K., Priebe G.P. (2022). Strategies to Prevent Ventilator-Associated Pneumonia, Ventilator-Associated Events, and Nonventilator Hospital-Acquired Pneumonia in Acute-Care Hospitals: 2022 Update. Infect. Control Hosp. Epidemiol..

[109] de Jong A., Pirracchio R., Kimmoun A. (2026). Checking Methodology and Statistics When Reading an Observational Study. Intensive Care Med..

[110] Fu E.L. (2023). Target Trial Emulation to Improve Causal Inference from Observational Data: What, Why, and How?. J. Am. Soc. Nephrol..

[111] Hernán M.A., Wang W., Leaf D.E. (2022). Target Trial Emulation: A Framework for Causal Inference From Observational Data. JAMA.

[112] Sinha P., Calfee C.S. (2019). Phenotypes in Acute Respiratory Distress Syndrome: Moving towards Precision Medicine. Curr. Opin. Crit. Care.

[113] Antcliffe D.B., Burrell A., Boyle A.J., Gordon A.C., McAuley D.F., Silversides J. (2025). Sepsis Subphenotypes, Theragnostics and Personalized Sepsis Care. Intensive Care Med..

[114] Ng J.H., Abdullah M.M., Abdel-Rahman E.M. (2025). Holistic Patient-Centered Outcomes in Post-Acute Kidney Injury Care: Physical, Emotional, Cognitive, and Social Outcomes. Adv. Kidney Dis. Health.

