# Supplementary material for: Ventilator-Associated Pneumonia: The Role of Kidney–Lung Crosstalk and Renal Replacement Therapy, a Narrative Review
Source: J Clin Med. 2026 Sep 29;15(19):7570. doi: 10.3390/jcm15197570 (PMC13666493; doi:10.3390/jcm15197570)

Supplementary Table S1 — Fluid resuscitation strategies and renal outcomes in the ICU

Study Year	Design	Population	Intervention	Main renal and respiratory outcomes	Limitations	Key message
Acute respiratory distress syndrome						
**FACTT Wiedemann et al.** 2006 [1]	Randomised trial	1,000 patients ARDS Multicentre, USA	Conservative strategy (CVP target < 4 mmHg) vs liberal strategy (CVP target 10–14 mmHg)	Ventilator-free days at day 28 significantly higher in the conservative group (15 vs 13 days, p = 0.04). Trend towards reduced dialysis use at day 60 (10% vs 14%, p = 0.06). No worsening of renal function in the conservative group. No difference in 60-day mortality.	Overall fluid strategy Renal endpoint secondary No VAP analysis	Landmark trial: conservative fluid management protects the lung without harming the kidney
Septic shock						
**RIFTS Corl et al.** 2019 [2]	Pilot randomised trial	109 patients Severe sepsis and septic shock Early phase (72 h) Single centre, USA	Restrictive strategy (total fluid cap 60 mL/kg over 72 h) vs usual care in the early phase of sepsis	Cumulative fluid balance at 72 h significantly lower in the restrictive group (p < 0.01). AKI or RRT requirement: no significant difference between groups (pilot trial underpowered for renal endpoints). Non-significant trend favouring the restrictive group for in-hospital mortality.	Pilot trial Single centre Underpowered for renal endpoints	Feasibility of early fluid restriction demonstrated in sepsis — renal outcomes inconclusive
**CLASSIC Meyhoff et al.** 2022 [3]	Randomised trial	1,554 patients Septic shock 31 European centres	Restrictive strategy (strict criteria for fluid boluses) vs standard care after initial resuscitation	No difference in 90-day mortality (42.3% vs 42.1%, p = 0.96). No reduction in severe AKI (KDIGO stage 3). Days alive without life support at 90 days similar between groups.	Post-resuscitation phase only Renal endpoint secondary	Restrictive strategy without renal or survival benefit in septic shock during the consolidation phase
**CLOVERS NHLBI PETAL Network** 2023 [4]	Randomised trial	1,563 patients Septic hypotension 60 centres, USA	Restrictive strategy (vasopressor-first approach) vs liberal strategy (fluid-first approach) in the early phase	No difference in 90-day mortality (14.0% vs 14.9%, p = 0.61). No difference in RRT-free days at day 28. Post-hoc analysis in patients with advanced CKD (eGFR < 30): favourable signal for mortality under restrictive strategy (HR 0.50 [0.29–0.85]).	Renal endpoint secondary Post-hoc signal, not pre-specified	No overall renal benefit. Post-hoc protective signal in patients with advanced CKD.
Established AKI						
**REVERSE-AKI Vaara et al.** 2021 [5]	Pilot randomised trial	100 patients Established euvolaemic AKI 7 centres, 5 countries	Restrictive strategy (neutral or negative fluid balance target) vs usual care	Significant reduction in cumulative fluid balance at 72 hours (difference of 1,148 mL, p = 0.033). Reduction in RRT requirement in adjusted analysis (13% vs 30%). No difference in AKI duration.	Pilot trial Limited sample size Adjusted analysis only	Only trial dedicated to fluid restriction in established AKI — favourable renal signal to be confirmed
**REDUCE Naorungroj et al.** 2025 [6]	Pilot randomised trial	Approximately 100 patients Mixed ICU population (sepsis, ARDS, post-operative)	Early fluid restriction and de-resuscitation (negative or neutral balance from day 1) vs usual care	Fluid balance significantly lower in the restrictive group. AKI: no significant difference between groups. RRT requirement: no significant difference. Non-significant trend favouring the restrictive group for 30-day mortality (12% vs 30%, p = 0.05).	Pilot trial Very limited sample size Heterogeneous population	Feasibility of early de-resuscitation demonstrated — renal benefit not yet established

Abbreviations: AKI, acute kidney injury; ARDS, acute respiratory distress syndrome; CKD, chronic kidney disease; CVP, central venous pressure; eGFR, estimated glomerular filtration rate; FO, fluid overload; ICU, intensive care unit; KDIGO, Kidney Disease: Improving Global Outcomes; RRT, renal replacement therapy.

1. National Heart, Lung, and Blood Institute Acute Respiratory Distress Syndrome (ARDS) Clinical Trials Network; Wiedemann, H.P.; Wheeler, A.P.; Bernard, G.R.; Thompson, B.T.; Hayden, D.; deBoisblanc, B.; Connors, A.F.; Hite, R.D.; Harabin, A.L. Comparison of Two Fluid-Management Strategies in Acute Lung Injury. *N. Engl. J. Med.* **2006**, *354*, 2564–2575, doi:10.1056/NEJMoa062200.
2. Corl, K.A.; Prodromou, M.; Merchant, R.C.; Gareen, I.; Marks, S.; Banerjee, D.; Amass, T.; Abbasi, A.; Delcompare, C.; Palmisciano, A.; et al. The Restrictive IV Fluid Trial in Severe Sepsis and Septic Shock (RIFTS): A Randomized Pilot Study. *Crit. Care Med.* **2019**, *47*, 951–959, doi:10.1097/CCM.0000000000003779.
3. Meyhoff, T.S.; Hjortrup, P.B.; Wetterslev, J.; Sivapalan, P.; Laake, J.H.; Cronhjort, M.; Jakob, S.M.; Cecconi, M.; Nalos, M.; Ostermann, M.; et al. Restriction of Intravenous Fluid in ICU Patients with Septic Shock. *N. Engl. J. Med.* **2022**, *386*, 2459–2470, doi:10.1056/NEJMoa2202707.
4. National Heart, Lung, and Blood Institute Prevention and Early Treatment of Acute Lung Injury Clinical Trials Network; Shapiro, N.I.; Douglas, I.S.; Brower, R.G.; Brown, S.M.; Exline, M.C.; Ginde, A.A.; Gong, M.N.; Grissom, C.K.; Hayden, D.; et al. Early Restrictive or Liberal Fluid Management for Sepsis-Induced Hypotension. *N. Engl. J. Med.* **2023**, *388*, 499–510, doi:10.1056/NEJMoa2212663.
5. Vaara, S.T.; Ostermann, M.; Bitker, L.; Schneider, A.; Poli, E.; Hoste, E.; Fierens, J.; Joannidis, M.; Zarbock, A.; van Haren, F.; et al. Restrictive Fluid Management versus Usual Care in Acute Kidney Injury (REVERSE-AKI): A Pilot Randomized Controlled Feasibility Trial. *Intensive Care Med.* **2021**, *47*, 665–673, doi:10.1007/s00134-021-06401-6.
6. Naorungroj, T.; Prajantasen, U.; Sanla-ead, T.; Viarasilpa, T.; Tongyoo, S. Restrictive Fluid Management with Early De-Escalation versus Usual Care in Critically Ill Patients (Reduce Trial): A Feasibility Randomized Controlled Trial. *Crit. Care* **2025**, *29*, 405, doi:10.1186/s13054-025-05624-z.

Supplementary Table S2 — Immunomodulatory agents and renal outcomes in the ICU: corticosteroids, anti-IL-6 and JAK inhibitors in CAP, ARDS and COVID-19

Study Year	Design	Population	Intervention	Main renal outcomes	Limitations	Key message
Severe community-acquired pneumonia						
**CAPE CODE Dequin et al.** 2023 [1]	Randomised trial Phase 3	795 patients Severe community-acquired pneumonia Multicentre, France	Hydrocortisone 200 mg/day as continuous infusion vs placebo (8 to 14 days total)	28-day mortality significantly reduced (6.2% vs 11.9%, HR 0.59 [0.40–0.86]). Renal data not reported as a primary endpoint. Parallel improvement in organ failure-free days, suggesting an indirect renal benefit mediated by reduced systemic inflammation.	Renal endpoint not isolated Trial stopped early	Significant reduction in mortality — indirect renal benefit suggested by reduction in organ failures
**ESCAPe Meduri et al.** 2022 [2]	Randomised trial	586 patients Severe CAP requiring ICU admission (onset within 72–96 h)	Methylprednisolone 40 mg bolus then 40 mg/day for 7 days, tapering over 20 days total, vs placebo	60-day mortality non-significant (OR 0.90 [0.57–1.40]). Reduction in ventilator days (p = 0.021). Individualised renal data not published. Negative trial on the primary endpoint with methodological differences from CAPE COD (fixed-duration protocol, methylprednisolone vs tailored hydrocortisone).	Negative primary endpoint Individualised renal data unavailable	Negative trial on mortality — ventilatory benefit without individualised renal data
**Torres et al.** 2015 [3]	Randomised trial	120 patients Severe CAP CRP > 150 mg/L	Methylprednisolone 0.5 mg/kg every 12 h for 5 days vs placebo	Treatment failure at 72 days significantly reduced (13% vs 31%, p = 0.02). Composite organ failures (OR 0.34 [0.14–0.87]). Isolated AKI data not reported separately.	Small sample size Renal endpoint not isolated	Significant benefit in patients with elevated CRP — composite organ failures reduced but renal endpoint not isolated
Acute respiratory distress syndrome						
**ARDSNet LaSRS Steinberg et al.** 2006 [4]	Randomised trial	180 patients Persistent ARDS (> 7 days) Multicentre, USA	Methylprednisolone 2 mg/kg/day for 14 days then taper vs placebo	Kidney failure-free days at day 28 significantly higher in the treatment group (22 vs 18 days, p = 0.04). No difference in 60-day mortality. Harmful signal observed when treatment was initiated after day 14.	Harmful signal if late initiation Mortality not improved	Only trial with a significant isolated renal endpoint in ARDS — renal benefit without mortality benefit
**Meduri et al.** 2007 [5]	Randomised trial	91 patients Early severe ARDS (< 72 h)	Methylprednisolone 1 mg/kg/day as continuous infusion for 21 days (progressive taper) vs placebo	Kidney failure-free days higher in the treatment group (24.3 vs 18.7 days, p = 0.015). Improvement in global organ failure score (p < 0.001). Positive renal signal consistent with reduction of systemic inflammation.	Small sample size Renal endpoint secondary	Positive signal on kidney failure-free days in early severe ARDS
Severe COVID-19						
**RECOVERY Dexamethasone Horby et al.** 2021 [6]	Randomised trial Platform	6,425 patients Hospitalised COVID-19 (29% on invasive MV) Multicentre, UK	Dexamethasone 6 mg/day orally or IV for up to 10 days vs usual care	RRT use at day 28 significantly reduced in patients on respiratory support (5% vs 7%, pre-specified subsidiary endpoint). 28-day mortality reduced (RR 0.83 [0.75–0.93], p < 0.001). Benefit concentrated in patients on oxygen or mechanical ventilation.	Open-label trial Renal endpoint subsidiary	Most robust renal signal among COVID-19 trials — significant reduction in RRT use alongside mortality reduction
**RECOVERY Tocilizumab RECOVERY Group** 2021 [7]	Randomised trial Platform	4,116 patients Hospitalised COVID-19 with hypoxaemia and inflammation (82% on corticosteroids)	Tocilizumab IV 400–800 mg (1 to 2 doses) vs usual care	Dialysis or haemofiltration at day 28: 5% vs 7% favouring tocilizumab (pre-specified subsidiary endpoint). 28-day mortality reduced (RR 0.85 [0.76–0.94], p = 0.0028). Additive benefits to dexamethasone.	Open-label trial Renal endpoint subsidiary	Reduction in dialysis requirement consistent with mortality reduction — additive benefits to dexamethasone
**RECOVERY Baricitinib RECOVERY Group** 2022 [8]	Randomised trial Platform	8,156 patients Hospitalised COVID-19 on oxygen Multicentre, UK	Baricitinib 4 mg/day orally for up to 10 days vs usual care	28-day mortality significantly reduced (12% vs 14%, RR 0.87 [0.77–0.98], p = 0.026). RRT or dialysis at day 28 (pre-specified subsidiary endpoint): no significant difference between groups. Dose adjustment required in patients with AKI (creatinine clearance < 60 mL/min).	Open-label trial Renal endpoint non-significant Dose adjustment required in AKI	Mortality benefit without significant renal effect — mandatory dose adjustment in AKI

Abbreviations: AKI, acute kidney injury; ARDS, acute respiratory distress syndrome; CAP, community-acquired pneumonia; CRP, C-reactive protein; ICU, intensive care unit; IL-6, interleukin-6; IV, intravenous; MV, mechanical ventilation; OR, odds ratio; RRT, renal replacement therapy; RR, rate ratio.

1. Dequin, P.-F.; Meziani, F.; Quenot, J.-P.; Kamel, T.; Ricard, J.-D.; Badie, J.; Reignier, J.; Heming, N.; Plantefève, G.; Souweine, B.; et al. Hydrocortisone in Severe Community-Acquired Pneumonia. *N. Engl. J. Med.* **2023**, *388*, 1931–1941, doi:10.1056/NEJMoa2215145.
2. Meduri, G.U.; Shih, M.-C.; Bridges, L.; Martin, T.J.; El-Solh, A.; Seam, N.; Davis-Karim, A.; Umberger, R.; Anzueto, A.; Sriram, P.; et al. Low-Dose Methylprednisolone Treatment in Critically Ill Patients with Severe Community-Acquired Pneumonia. *Intensive Care Med.* **2022**, *48*, 1009–1023, doi:10.1007/s00134-022-06684-3.
3. Torres, A.; Sibila, O.; Ferrer, M.; Polverino, E.; Menendez, R.; Mensa, J.; Gabarrús, A.; Sellarés, J.; Restrepo, M.I.; Anzueto, A.; et al. Effect of Corticosteroids on Treatment Failure among Hospitalized Patients with Severe Community-Acquired Pneumonia and High Inflammatory Response: A Randomized Clinical Trial. *JAMA* **2015**, *313*, 677–686, doi:10.1001/jama.2015.88.
4. Steinberg, K.P.; Hudson, L.D.; Goodman, R.B.; Hough, C.L.; Lanken, P.N.; Hyzy, R.; Thompson, B.T.; Ancukiewicz, M.; National Heart, Lung, and Blood Institute Acute Respiratory Distress Syndrome (ARDS) Clinical Trials Network Efficacy and Safety of Corticosteroids for Persistent Acute Respiratory Distress Syndrome. *N. Engl. J. Med.* **2006**, *354*, 1671–1684, doi:10.1056/NEJMoa051693.
5. Meduri, G.U.; Golden, E.; Freire, A.X.; Taylor, E.; Zaman, M.; Carson, S.J.; Gibson, M.; Umberger, R. Methylprednisolone Infusion in Early Severe ARDS: Results of a Randomized Controlled Trial. *Chest* **2007**, *131*, 954–963, doi:10.1378/chest.06-2100.
6. RECOVERY Collaborative Group; Horby, P.; Lim, W.S.; Emberson, J.R.; Mafham, M.; Bell, J.L.; Linsell, L.; Staplin, N.; Brightling, C.; Ustianowski, A.; et al. Dexamethasone in Hospitalized Patients with Covid-19. *N. Engl. J. Med.* **2021**, *384*, 693–704, doi:10.1056/NEJMoa2021436.
7. Abani, O.; Abbas, A.; Abbas, F.; Abbas, M.; Abbasi, S.; Abbass, H.; Abbott, A.; Abdallah, N.; Abdelaziz, A.; Abdelfattah, M.; et al. Tocilizumab in Patients Admitted to Hospital with COVID-19 (RECOVERY): A Randomised, Controlled, Open-Label, Platform Trial. *The Lancet* **2021**, *397*, 1637–1645, doi:10.1016/S0140-6736(21)00676-0.
8. RECOVERY Collaborative Group Baricitinib in Patients Admitted to Hospital with COVID-19 (RECOVERY): A Randomised, Controlled, Open-Label, Platform Trial and Updated Meta-Analysis. *Lancet* **2022**, *400*, 359–368, doi:10.1016/S0140-6736(22)01109-6.

Supplementary Table S3 — Sodium bicarbonate in the ICU (BICAR-ICU / BICARICU-2): impact on respiratory function and the kidney-lung crosstalk

StudyYear	DesignPopulation	Intervention	Primary outcomes —kidney-lung link	Secondary respiratoryoutcomes	Main limitations	Clinical conclusion
**BICAR-ICU Jaber et al.**2018 [1]	Randomised trialMulticentreFrance (26 ICUs)n = 389pH ≤ 7.20PaCO2 ≤ 45 mmHgBicarbonate ≤ 20 mmol/LSOFA score ≥ 482% on invasive MV80% on vasopressors51% septic shock	Sodium bicarbonate 4.2% IV infusion (target pH ≥ 7.30, maximum 1 L/24 h)vsUsual careRandomisation within 48 hours of ICU admission, stratified by presence of AKI AKIN stages 2–3	Primary endpoint (death at day 28 or ≥ 1 organ failure at day 7): 71% vs 66% — non-significant (p = 0.24) in the overall populationSubgroup AKI AKIN stages 2–3 (n = 182): 28-day mortality 63% vs 46% (p = 0.017) — SIGNIFICANTMV duration: not reported sequentially in the overall population	No difference in RRT-free days in survivorsFluid overload: +1 L in the bicarbonate group — theoretical risk of pulmonary oedema not confirmedPaO2/FiO2 ratio: threshold < 200 retained as urgent RRT indication but no longitudinal follow-upNo data on ventilator-free days or early extubation24% of the control group received bicarbonate (contamination)	Open-label trial (blood gas masking not feasible)24% contamination in the control armSubgroup result pre-specified but not confirmatoryVolume of bicarbonate administered potentially worsening fluid overload — uncontrolled biasNo kidney-lung mediation analysis	Neutral in the overall population. Positive signal in the severe AKI subgroup (AKIN stages 2–3) with reduced mortality and organ failures. Bicarbonate appears to remove a metabolic trigger (acidaemia) delaying RRT initiation, with a possible indirect impact on dialytic fluid burden and MV duration.Subgroup result: hypothesis-generating
**BICAR-ICU-2 Jung et al.**2025 [2]	Randomised trialMulticentreFrance (43 ICUs)n = 627pH ≤ 7.20Bicarbonate ≤ 20 mmol/LPaCO2 ≤ 45 mmHgKDIGO stages 2–375% on invasive MV80% on vasopressorsMedian age 67 years	Sodium bicarbonate 4.2% IV infusion (target pH ≥ 7.30, median 750 mL over 24 h)vsUsual care without bicarbonate1:1 randomisation by minimisation	90-day mortality (primary endpoint): 62.1% vs 61.7% — non-significant (p = 0.91)RRT use at day 28 (secondary endpoint): 35% vs 50% — SIGNIFICANT (p < 0.001)Median time to RRT initiation: 31 h vs 15.5 h — SIGNIFICANTMV duration: no difference between groups	No effect of bicarbonate on:— Invasive MV duration— Vasopressor duration— ICU or hospital length of stay— Organ failure score at day 7— Overall adverse eventsCatheter-related infections: lower in the bicarbonate group (4% vs 9%)Fluid balance: similar between groups despite 750 mL additional fluid	Open-label trial (unavoidable given the nature of the intervention)Powered for mortality — potentially underpowered to detect MV effects (secondary endpoint)French population only (limited generalisability)No mediation analysis: bicarbonate → reduced RRT → respiratory impactBeneficial signal from BICAR-ICU severe AKI subgroup not replicated (BICARICU-2 enrolled only stage 2–3 AKI patients)	Confirms BICAR-ICU: bicarbonate does not modify mortality or respiratory outcomes in severe AKI with acidaemia. The robust reduction in RRT use (minus 15 absolute percentage points) does not translate into MV or survival benefit — suggesting that RRT initiation timing is driven by arbitrary metabolic thresholds rather than by worsening pulmonary function.Uncoupling: RRT reduction without respiratory benefit

. ***Abbreviations:*** *AKI, acute kidney injury; AKIN, Acute Kidney Injury Network classification; ICU, intensive care unit; IV, intravenous; KDIGO, Kidney Disease: Improving Global Outcomes classification; MV, mechanical ventilation; $PaCO_2$, arterial partial pressure of carbon dioxide; PaO_2/FiO_2, arterial oxygen tension-to-inspired oxygen fraction ratio; RRT, renal replacement therapy; SOFA, Sequential Organ Failure Assessment score.*

1. Jaber, S.; Paugam, C.; Futier, E.; Lefrant, J.-Y.; Lasocki, S.; Lescot, T.; Pottecher, J.; Demoule, A.; Ferrandière, M.; Asehnoune, K.; et al. Sodium Bicarbonate Therapy for Patients with Severe Metabolic Acidaemia in the Intensive Care Unit (BICAR-ICU): A Multicentre, Open-Label, Randomised Controlled, Phase 3 Trial. *Lancet* **2018**, *392*, 31–40, doi:10.1016/S0140-6736(18)31080-8.
2. Jung, B.; Jabaudon, M.; De Jong, A.; Bitker, L.; Audard, J.; Klouche, K.; Sarton, B.; Guitton, C.; Lasocki, S.; Rieu, B.; et al. Sodium Bicarbonate for Severe Metabolic Acidemia and Acute Kidney Injury: The BICARICU-2 Randomized Clinical Trial. *JAMA* **2025**, *334*, 2000–2010, doi:10.1001/jama.2025.20231.

Supplementary Table S4 — Timing of RRT initiation: impact on respiratory function

StudyYear	DesignPopulation	Intervention(early vs delayed)	Respiratoryoutcomes	Secondary respiratoryoutcomes	Main limitations	Clinical conclusion
A — Trials in favour of early RRT						
**ELAIN Zarbock et al.**2016 [1]	Randomised trialSingle centreGermanyn = 23195% surgical patientsKDIGO stage 2with NGAL > 150 ng/mL	Early: RRT within 8 hours of KDIGO stage 2vsDelayed: RRT within 12 hours of KDIGO stage 3 or never(90.8% of the delayed arm ultimately received RRT)	Median duration of mechanical ventilation: 125.5 h vs 181.0 h (p = 0.002) — SIGNIFICANT90-day mortality: 39.3% vs 53.6% (HR 0.66 [0.45–0.97], p = 0.02) — SIGNIFICANT	IL-6 and IL-8 at 24 hours significantly lower in the early armHospital length of stay: 51 vs 82 days (p < 0.001)Ventilator-free days at day 28: not reportedPaO2/FiO2 ratio: not reported sequentially	Single centreMajority surgical population (major selection bias, not generalisable to medical ICU patients)Initiation from KDIGO stage 2, vs stage 3 in all other trials	Only trial demonstrating a benefit of early RRT on MV duration and mortality. Reduction in ventilation duration of 55 hours (possibly attributable to early clearance of inflammatory mediators and fluid overload).
B — Trials neutral on respiratory function						
**AKIKI Gaudry et al.**2016 [2]	Randomised trialMulticentreFrance (31 ICUs)n = 62080% sepsis or septic shockKDIGO stage 3On MV or vasopressors	Early: RRT within 3 hoursvsDelayed: RRT only if urgent criterion met (hypoxaemic pulmonary oedema, potassium > 6 mmol/L, pH < 7.15, urea > 40 mmol/L or prolonged oliguria)49% of the delayed arm never received RRT	60-day mortality: 48.5% vs 49.7% — non-significant (p = 0.79)MV duration at day 28: not reported as a primary endpointHypoxaemic pulmonary oedema with FiO2 > 50%: among the triggering criteria in the delayed arm — not analysed separately	Ventilator-free days at day 28: not reportedPaO2/FiO2 ratio: not reportedDialysis-free survival: better in the delayed arm (49% never received RRT)	Open-label trialNo MV data as a secondary endpointPopulation heterogeneity (MV and vasopressors)Hypoxaemic pulmonary oedema as a delayed trigger but incidence not analysed	No benefit of early RRT on mortality. A watchful waiting approach is safe and avoids RRT in nearly half of patients.
**IDEAL-ICU Barbar et al.**2018 [3]	Randomised trialMulticentreFrance (29 ICUs)n = 488100% septic shockAKI RIFLE stage FOn vasopressors	Early: RRT within 12 hours of RIFLE stage FvsDelayed: RRT after 48 hours of RIFLE stage F or if urgent criterion met29% of the delayed arm did not receive RRT (spontaneous recovery)	90-day mortality: 58% vs 54% — non-significant (p = 0.38)Ventilator-free days at day 28 (median): 2 vs 3 days — non-significantPulmonary oedema among urgent triggers in the delayed arm: 4% vs < 1% in the early arm	Vasopressor-free days: 16 vs 17 — non-significantHospital length of stay: 23 days in both groupsPaO2/FiO2 ratio: not reported sequentially	Early termination for futility (488/864 planned patients enrolled)Reduced power for secondary endpointsVery high mortality (> 55%) limiting detectability of any MV effect	No benefit of early RRT in septic shock. Near-identical ventilator-free days suggest that fluid management or cytokine clearance does not impact MV duration in this context.
**STARRT-AKI The STARRT-AKIInvestigators**2020 [4]	Randomised trialInternational multicentre168 ICUs, 15 countriesn = 2,927AKI KDIGO stages 2–3On MV and/or vasopressorsHeterogeneous population(medical and surgical)	Accelerated: RRT within 12 hours of eligibilityvsStandard: RRT deferred until at least one urgent criterion met (potassium ≥ 6, bicarbonate ≤ 10, PaO2/FiO2 < 200, or persistent AKI > 72 hours)40% of the standard arm did not receive RRT	90-day mortality: 43.9% vs 43.7% — non-significant (RR 1.00 [0.93–1.09])Ventilator-free days at day 28 (median): 13 vs 12 — non-significantPaO2/FiO2 < 200: used as a trigger in the standard arm but without longitudinal follow-up	Vasopressor-free days at day 28: 21 vs 20 — non-significantAdverse events: higher in the accelerated arm	Open-label trialPopulation heterogeneity and inter-country practice variationNo sequential analysis of oxygenation parametersPotential confounding: PaO2/FiO2 < 200 as a trigger in the standard arm	Largest RCT on RRT timing. Neutral on all endpoints including MV duration. Convergent with AKIKI and IDEAL-ICU.
**AKIKI-2 Gaudry et al.**2021 [5]	Randomised trialMulticentreFrance (39 ICUs)n = 278KDIGO stage 3 prolonged AKI(oliguria > 72 h and/orurea > 40 mmol/L)On MV or vasopressorsNo urgent indication	Standard: RRT within 12 hours of meeting AKIKI delayed-arm criteriavsEven more delayed: RRT deferred until urgent indication or urea > 50 mmol/L (oliguria alone insufficient)	RRT-free days at day 28 (primary endpoint): 12 vs 10 — non-significantVentilator-free days at day 28: no difference between groupsMortality: signal of excess mortality in the very delayed arm (adjusted HR 1.65) — unconfirmed (underpowered)	No difference in ICU length of stay or renal recoveryMetabolic acidosis complications more frequent in the very delayed arm	Small sample size (278/810 planned patients)Pre-selection of prolonged KDIGO stage 3 AKI — particularly severe population, not generalisableExcess mortality signal unconfirmed	Defines a lower boundary for acceptable delay: prolonging beyond the AKIKI standard delay provides no additional benefit and exposes patients to a mortality signal. No impact on MV duration.

Abbreviations: AKI, acute kidney injury; FiO_2, fraction of inspired oxygen; HR, hazard ratio; ICU, intensive care unit; KDIGO, Kidney Disease: Improving Global Outcomes; MV, mechanical ventilation; NGAL, neutrophil gelatinase-associated lipocalin; PaO_2/FiO_2, arterial oxygen tension-to-inspired oxygen fraction ratio; RIFLE, Risk, Injury, Failure, Loss, End-stage kidney disease; RR, relative risk; RRT, renal replacement therapy.

1. Zarbock, A.; Kellum, J.A.; Schmidt, C.; Van Aken, H.; Wempe, C.; Pavenstädt, H.; Boanta, A.; Gerß, J.; Meersch, M. Effect of Early vs Delayed Initiation of Renal Replacement Therapy on Mortality in Critically Ill Patients With Acute Kidney Injury: The ELAIN Randomized Clinical Trial. *JAMA* **2016**, *315*, 2190–2199, doi:10.1001/jama.2016.5828.
2. Gaudry, S.; Hajage, D.; Schortgen, F.; Martin-Lefevre, L.; Pons, B.; Boulet, E.; Boyer, A.; Chevrel, G.; Lerolle, N.; Carpentier, D.; et al. Initiation Strategies for Renal-Replacement Therapy in the Intensive Care Unit. *N. Engl. J. Med.* **2016**, *375*, 122–133, doi:10.1056/NEJMoa1603017.
3. Barbar, S.D.; Clere-Jehl, R.; Bourredjem, A.; Hernu, R.; Montini, F.; Bruyère, R.; Lebert, C.; Bohé, J.; Badie, J.; Eraldi, J.-P.; et al. Timing of Renal-Replacement Therapy in Patients with Acute Kidney Injury and Sepsis. *N. Engl. J. Med.* **2018**, *379*, 1431–1442, doi:10.1056/NEJMoa1803213.
4. STARRT-AKI Investigators; Canadian Critical Care Trials Group; Australian and New Zealand Intensive Care Society Clinical Trials Group; United Kingdom Critical Care Research Group; Canadian Nephrology Trials Network; Irish Critical Care Trials Group; Bagshaw, S.M.; Wald, R.; Adhikari, N.K.J.; Bellomo, R.; et al. Timing of Initiation of Renal-Replacement Therapy in Acute Kidney Injury. *N. Engl. J. Med.* **2020**, *383*, 240–251, doi:10.1056/NEJMoa2000741.
5. Gaudry, S.; Hajage, D.; Martin-Lefevre, L.; Lebbah, S.; Louis, G.; Moschietto, S.; Titeca-Beauport, D.; Combe, B.L.; Pons, B.; de Prost, N.; et al. Comparison of Two Delayed Strategies for Renal Replacement Therapy Initiation for Severe Acute Kidney Injury (AKIKI 2): A Multicentre, Open-Label, Randomised, Controlled Trial. *Lancet* **2021**, *397*, 1293–1300, doi:10.1016/S0140-6736(21)00350-0.

Supplementary Figure S1 — Flow-chart of the study selection

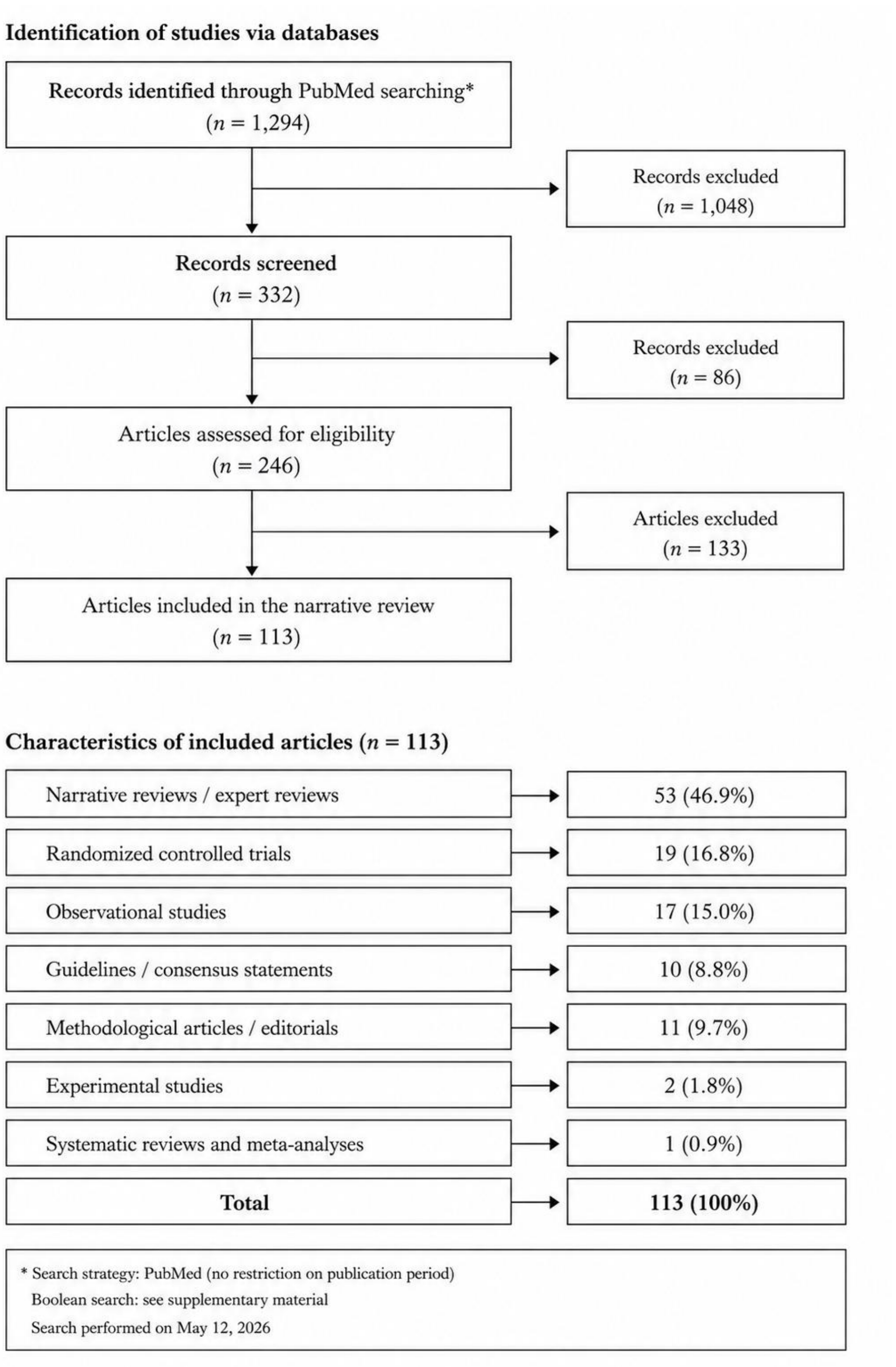

Supplement: Supplementary file 1 [file jcm-15-07570-s001.zip › jcm-4416592-supplementary.pdf]
